# SRPK1/2 and PP1α exert opposite functions by modulating SRSF1-guided *MKNK2* alternative splicing in colon adenocarcinoma

**DOI:** 10.1186/s13046-021-01877-y

**Published:** 2021-02-18

**Authors:** Hongda Liu, Zheng Gong, Kangshuai Li, Qun Zhang, Zekuan Xu, Yunfei Xu

**Affiliations:** 1 Department of General Surgery, The First Affiliated Hospital of Nanjing Medical University, 300 Guangzhou Road, Nanjing, 210029 China; 2 The Jackson Laboratory, Bar Harbor, ME 04609 USA; 3 Department of General Surgery, Qilu Hospital of Shandong University, Jinan, 250012 China; 4 Department of Respiratory Medicine, The First Affiliated Hospital of Nanjing Medical University, Nanjing, 210029 China

**Keywords:** Alternative splicing, Colon adenocarcinoma, *MKNK2*, SRSF1, SRPK1/2

## Abstract

**Background:**

The Mnk2 kinase, encoded by *MKNK2* gene, plays critical roles in MAPK signaling and was involved in oncogenesis. Human *MKNK2* pre-mRNA can be alternatively spliced into two splicing isoforms, the *MKNK2a* and *MKNK2b*, thus yielding Mnk2a and Mnk2b proteins with different domains. The involvement of Mnk2 alternative splicing in colon cancer has been implicated based on RNA-sequencing data from TCGA database. This study aimed at investigating the upstream modulators and clinical relevance of Mnk2 alternative splicing in colon adenocarcinoma (CAC).

**Methods:**

PCR, western blotting and immunohistochemistry (IHC) were performed to assess the expression of Mnk2 and upstream proteins in CAC. The function of Mnk2 and its regulators were demonstrated in different CAC cell lines as well as in xenograft models. Two independent cohorts of CAC patients were used to reveal the clinical significance of MKNK2 alternative splicing.

**Results:**

Comparing with adjacent nontumorous tissue, CAC specimen showed a decreased *MKNK2a* level and an increased *MKNK2b* level, which were correlated with *KRAS* mutation and tumor size. The SRSF1 (serine/arginine-rich splicing factor 1) was further confirmed to be the major splicing factor targeting *MKNK2* in CAC cells. Higher expression of SRPK1/2 or decreased activity of PP1α were responsible for enhancing SRSF1 phosphorylation and nucleus translocation, subsequently resulted in a switch of *MKNK2* alternative splicing.

**Conclusions:**

Our data showed that phosphorylation and subcellular localization of SRSF1 were balanced by SRPK1/2 and PP1α in CAC cells. High nucleus SRSF1 promoted *MKNK2* splicing into *MKNK2b* instead of *MNK2a*, consequently enhanced tumor proliferation.

**Supplementary Information:**

The online version contains supplementary material available at 10.1186/s13046-021-01877-y.

## Background

Colorectal cancer ranks the third on both malignant morbidity and cancer-related deaths worldwide [1]. Approximately 20% of colorectal cancer patients were diagnosed at an extensive-stage with distant distribution, and the 5-year survival rate is less than 15% [2]. As the most common histological subtype, colon adenocarcinoma (CAC) can be treated with surgical resection, adjuvant chemotherapy, and radiation treatment. Unfortunately, molecular understanding of CAC progression remains limited although its incidence and mortality rates have been declined during the past decades [3].

Numerous well-known kinase networks are considered to play roles in CAC pathogenesis, such as EGFR, MAPKs, c-Src, et al. The MAP kinase-interacting serine/threonine-protein kinases (Mnks), downstream of MAPKs, are protein kinases that can phosphorylate eIF4E and enhance oncogenic mRNA translation [4]. There are two members of Mnk family in human cells: Mnk1 and Mnk2. We have previously reported the clinical significance of Mnk1 in epithelial ovarian cancer [5]. Mnk2 has also been reported to be involved in malignancies such as prostate cancer and lung cancer [6, 7]. Mnk2a and Mnk2b are two protein isoforms that derived from the *MKNK2* pre-mRNA by alternative splicing, namely *MKNK2a* and *MKNK2b*, respectively [8]. Mnk2a contains a MAPK-binding site in its C-terminus while Mnk2b lacks it [9]. The specific role of Mnk2a seems ambiguous in tumorigenesis. On one hand, hyperactivation of Mnk2a can phosphorylate eIF4E thus exerts oncogenic effects [9]. One the other hand, Mnk2a directly interacts and phosphorylates p38α -MAPK, subsequently inducing cell death and suppressing Ras-induced transformation [10]. In contrast, Mnk2b is generally recognized as a pro-oncogenic kinase due to its deficiency in binding p38α -MAPK but reserves capacity of phosphorylating eIF4E [10].

According to TCGA database, the *MKNK2a*/*MKNK2b* ratio was reported to be downregulated in CAC tissues compared with nontumorous colon tissues [10], however its clinical significance and upstream regulators remain unknown. Here we discovered that SRSF1 (Serine and Arginine Rich Splicing Factor 1), an essential sequence specific splicing factor involved in pre-mRNA splicing, is the predominant splicing factor that promotes synthesis of *MKNK2b* instead of *MKNK2a*. Interestingly, SRSF1-guided *MKNK2a*-*MKNK2b* switch is dependent on its phosphorylation and subsequent nucleus transportation. Our data further revealed the antergic effects of SRPK1/2 (SR-specific protein kinase) and protein phosphatase 1α (PP1α) on directly modulating SRSF1 phosphorylation. Besides, SRPK1/2 and nucleus SRSF1 were verified to be independent prognostic factors for the clinical outcome of CAC patients from two retrospective cohorts. Finally, the tumor-related role of SRPK1/2, SRSF1, Mnk2b, and PP1α were validated in CAC cell lines as well as in xenograft models.

## Methods

### Cells and reagents

Human CAC cell lines including Caco-2, HT-29, HCT-116, SW480 and SW620 were purchased from the American Type Culture Collection (ATCC, Manassas, VA, USA). Nontumorous NCM460 colon epithelial cells were obtained from Jennio Biotechnology (Guangzhou, China). Cells were cultured in DMEM supplemented with 10% fetal bovine serum (FBS) and 1% penicillin/streptomycin under 5% CO2 at 37 °C atmosphere. The chemicals used in our study were listed in Supplementary Table S6.

### DNA constructs, siRNAs, shRNAs

pCDNA3.1-vector was obtained from Invitrogen. Full-length of *KRAS*-G12V cDNA was cut from pBabe K-Ras 12 V vector (Cat. #12544, Addgene) and cloned into pcDNA3.1-vector (Invitrogen) via BamH1 and Xba1 restriction sites, thus generating the pCDNA3.1-RAS-G12V construct. Other DNA constructs including pCDNA3.1-Mnk2a, pCDNA3.1-Mnk2b, pCDNA3.1-SRPK1-WT, pCDNA3.1-SRPK1-K109A, pCDNA3.1-SRPK2-WT, pCDNA3.1-SRPK2-K110A, pCDNA3.1-PP1α-WT, pCDNA3.1-PP1α-T320A, and pCDNA3.1-PP1α-H125A were generated by GenePharma (Shanghai, China). The siRNAs and shRNAs were listed in Supplementary Table S6.

Transient transfections were conducted by using Lipofectamine3000 Transfection Reagent (Cat. # L3000015, Thermo Fisher Scientific, Pittsburgh, PA, USA) according to the manufacturer’s instructions. Puromycin was used to select and maintain stable overexpression cells. Lentivirus infection of SRPK1 and SPRK2 shRNAs were conducted as previously described [11].

### Patients and tissue samples

Two independent cohorts of patients were retrospectively enrolled in this study. The primary cohort enrolled 32 CAC patients who underwent surgical treatment in Qilu Hospital of Shandong University (Jinan, China) from 2016 to 2017. The fresh-resected carcinoma tissues and paired adjacent nontumorous colon tissues were all frozen in liquid-nitrogen until experimental test. The validation cohort comprised of 100 CAC cases that underwent tumor resection in 2007, and the tissue specimens were formalin-fixed paraffin-embedded (#HColA180Su17, Shanghai Outdo Biotech, Shanghai, China). The patients in validation cohort were followed up until July 2015 (ranging 2–97 months). All the patients in primary cohort and validation cohort underwent R0 resection and classified with TNM stage I-IIIC at the time of surgical intervention according to the 7th AJCC/UICC TNM classification system. None of the patients in the two cohorts received any adjunctive therapy before tissue sample collection. The clinicopathological characteristics of the patients in two cohorts were summarized in Supplemental Table S1 and Supplemental Table S3, respectively. Informed consents were obtained from all patients or immediate relatives. All experiments were approved and supervised by the Ethics Committee of Qilu Hospital of Shandong University.

### Real-time PCR (RT-PCR) and real-time quantitative PCR (RT-qPCR)

Paired tumor and nontumorous specimens from primary cohort were subjected for RNA isolation and RT-qPCR using *GAPDH* as internal control. Briefly, total RNA was extracted from tissues and cells using TRIzol reagent. RNA was reversely transcribed into cDNA by Prime-Script RT Master Mix (TaKaRa, Kyoto, Japan). Quantitative polymerase chain reaction (qPCR) was conducted with SYBR Premix Ex Taq II kits (TaKaRa, Kyoto, Japan) as described before [12]. RT-PCR was performed using the one step RT-PCR kit (QIAGEN) following the manufacturer’s protocol. The primers used for RT-PCR and RT-qPCR were listed in Supplemental Table S6.

### Cell proliferation and colony formation

The proliferation assay colony formation assay were conducted as previously described [13, 14]. Proliferation capacity was assessed by 3-(4,5-dimethylthiazol-2-yl)-2,5-diphenyl-2H-tetrazolium bromide (MTT) assays at designated time points (6 h, 24 h, 48 h, 72 h, 96 h). Colonies were tested after incubation for 10 days in DMEM.

### RNA-binding protein immunoprecipitation (RIP)

The RIP assay was conducted by using an RNA-Binding Protein Immunoprecipitation Kit (Millipore) according to the manufacture’s procedure. The binding between the SRSF1 protein and *MKNK2a/2b*-mRNA was determined by RT-PCR and Western blotting of immunoprecipitated mixture.

### Sample lysis and immunoblotting

Tissue samples or cultured cells were lysed using RIPA lysis buffer (Cat. #P0013, Beyotime, Beijing, China) containing protease and phosphatase inhibitors for total protein test. For nucleus isolation, cells were processed using the Nucleus and Cytosol Protein Extraction Kit (Cat. #P0027, Beyotime) according to the manufacture’s instruction. The immunoblotting was conducted as described previously [14]. The antibodies used in our study were listed in Supplementary Table S6 at 1:1000 dilution.

### Immunohistochemical (IHC) staining

The IHC staining was performed as described before [14] using anti-SRSF1, anti-SRPK1, anti-SRPK2, anti-PP1α, anti-PP1α-p-Thr320 antibodies (Table S6) at 1:300 dilution. The staining results were semi-quantified by multiplying the positive percentage score (0, no positive stained cells; 1, 0–25% positive; 2, 26–50% positive; 3, 51–75% positive; 4, 76–100% positive) and staining intensity score (0, negative staining; 1, slightly yellow; 2, dark yellow; 3, yellow brown), ranging 0–12. As for the quantification of SRSF1 staining, the nucleus staining and cytosol staining were scored separately. Either the nucleus staining or the cytosol staining were scored as 0–12 as described above. The cut-off of IHC scores were obtained by using receiver operating characteristic (ROC) curves to distinguish high- or low-expression groups.

### Immunoprecipitation

Transfected cells were harvested and lysed with RIPA buffer. After protein quantification using the BCA kit (Thermo Fisher Scientific), equal amount of lysates were incubated with HA agarose beads overnight an 4 °C. The immunoprecipitated mixture were subjected to Western blotting after washing out the nonspecific bindings with RIPA buffer.

### GST-pull down

The purified GST protein was obtained from Prof. Jinpeng Sun in Shandong University (Jinan, China). The purified GST-tagged SRPK1 and GST-tagged SRPK2 proteins were purchased from Novus Biologicals (CO, USA). The His-SRSF1 protein was purchased from Creative Biomart (NY, USA). The GST-pull down assay was conducted as described before using GST agarose beads [15].

### In vitro phosphorylation test

Purified SRPK1 or SRPK2 proteins were incubated with SRSF1 proteins in kinase reaction buffer (25 mM Tris, pH 7.5, with 10 mM MgCl2, 2 mM DTT, 5 mM β-Glycerolphosphate, 0.1 mM Na3VO4, and 2 mM EGTA) containing 20 μM ATP for 30 min at 37 °C water bath. Reactions were then quenched by adding SDS loading buffer and subjected to western blot assay.

### Mice model and xenograft

Transfected SW480 cells (4 × 10^6^) were subcutaneously injected into six-week male Balb/c nude mice. Each group contained 5 mice. Tumor size was measured every 5 days using the following formula: V = (length×width^2^)/2. The mice were sacrificed on the 21st day after injection, and the xenograft tumors were removed and weighed as described before [13]. The Animal Welfare Committee of Shandong University approved all procedures involving animals.

### Statistics

Data are presented as the mean ± standard deviation (SD). Statistical analyses were conducted using SPSS 20.0 Software. Correlations between mRNAs were analyzed using Spearman correlation test. Correlations between proteins and patients’ characteristics were analyzed using Chi-square test. Overall survival (OS) was assessed using the Kaplan-Meier method and significance was analyzed using the log-rank test. Multivariate cox regression analyses were carried out to identify independent prognostic factors. For the cellular and xenograft data, the difference between two groups was tested using Student’s t-test, while One-way ANOVA analysis was used to compare data among more than two groups. A value of *P* < 0.05 indicated a statistically significant result.

## Results

### *MKNK2* alternative splicing is altered in CAC tissues and affected by *KRAS* mutation

**Fig. 1:**
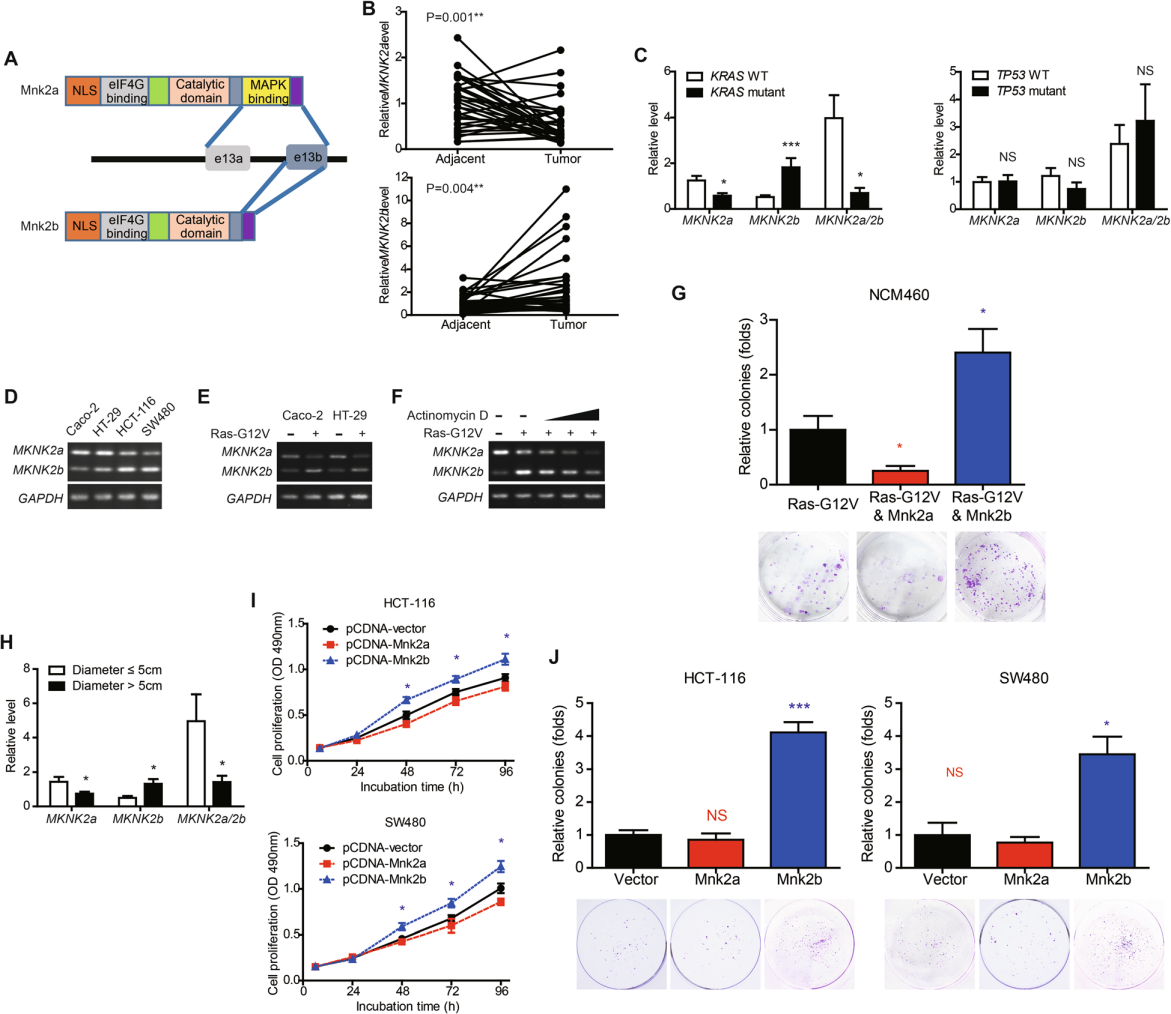
Alternative splicing of *MKNK2* in CAC tissues is correlated with oncogenic characteristics. **a** The schematic model of Mnk2 isoforms. *MKNK2a* and *MKNK2b* possess different exon 13. The C-terminus of Mnk2a is translated from exon13a and exon13b, therefore contains a MAPK binding motif and can bind with MAPK members. In contrast, Mnk2b is generated by an alternative 3′ splicing site between exon13a and exon13b, leading to the absence of MAPK binding motif. **b** RNA levels of *MKNK2a* and *MKNK2b* were tested via RT-qPCR in 32 pairs of CAC tumor tissues and adjacent nontumorous tissues. *P* value was based on paired Student’s t-test. **c** Clinical significance of *MKNK2* alternative splicing was evaluated by analyzing its correlation with *KRAS* and *TP53* mutations. **d** Expressing of *MKNK2a* and *MKNK2b* were tested in distinct CAC cell lines via RT-PCR. Caco-2 and HT-29 cells are documented with wild type *KRAS*, while HCT-116 and SW480 cells with mutated-*KRAS* (G13D and G12V mutation, respectively). **e** RAS-G12V transfecting resulted in decreased *MKNK2a* and increased *MKNK2b* in both Caco-2 and HT-29 cells. **f** Actinomycin D (20 nM, 80 nM, 200 nM) was used to assess whether the RAS-G12V-induced upregulation of *MKNK2b* was caused by enhanced RNA synthesis or attenuated RNA-damaging. Accordingly, Ras-G12V can enhance *MKNK2a-MKNK2b* switch while actinomycin D inhibited RNA synthesis. **g** Colony formation assays revealed that RAS-G12V-induced oncogenic transformation was inhibited by Mnk2a while enhanced by Mnk2b transfection. *P* value was based on unpaired Student’s t-test comparing with RAS-G12V group. (H) A lower *MKNK2a* level and a higher *MKNK2b* level were found in tumors with larger size (*n* = 20) comparing with the smaller ones (*n* = 12). *P* value was based on unpaired Student’s t-test. (I) MTT proliferation assays showed the oncogenic effect of Mnk2b on promoting tumor proliferation in both HCT-116 and SW480 cells. *P* value was based on unpaired Student’s t-test comparing with cells transfected with pcDNA-vector. (J) Overexpressing Mnk2b enhanced colony formation of the CAC cell line HCT-116 and SW480 cells. Colonies were counted after incubation for 10 days in DMEM. P value was based on unpaired Student’s t-test comparing with cells transfected with pcDNA-vector

**Fig. 2:**
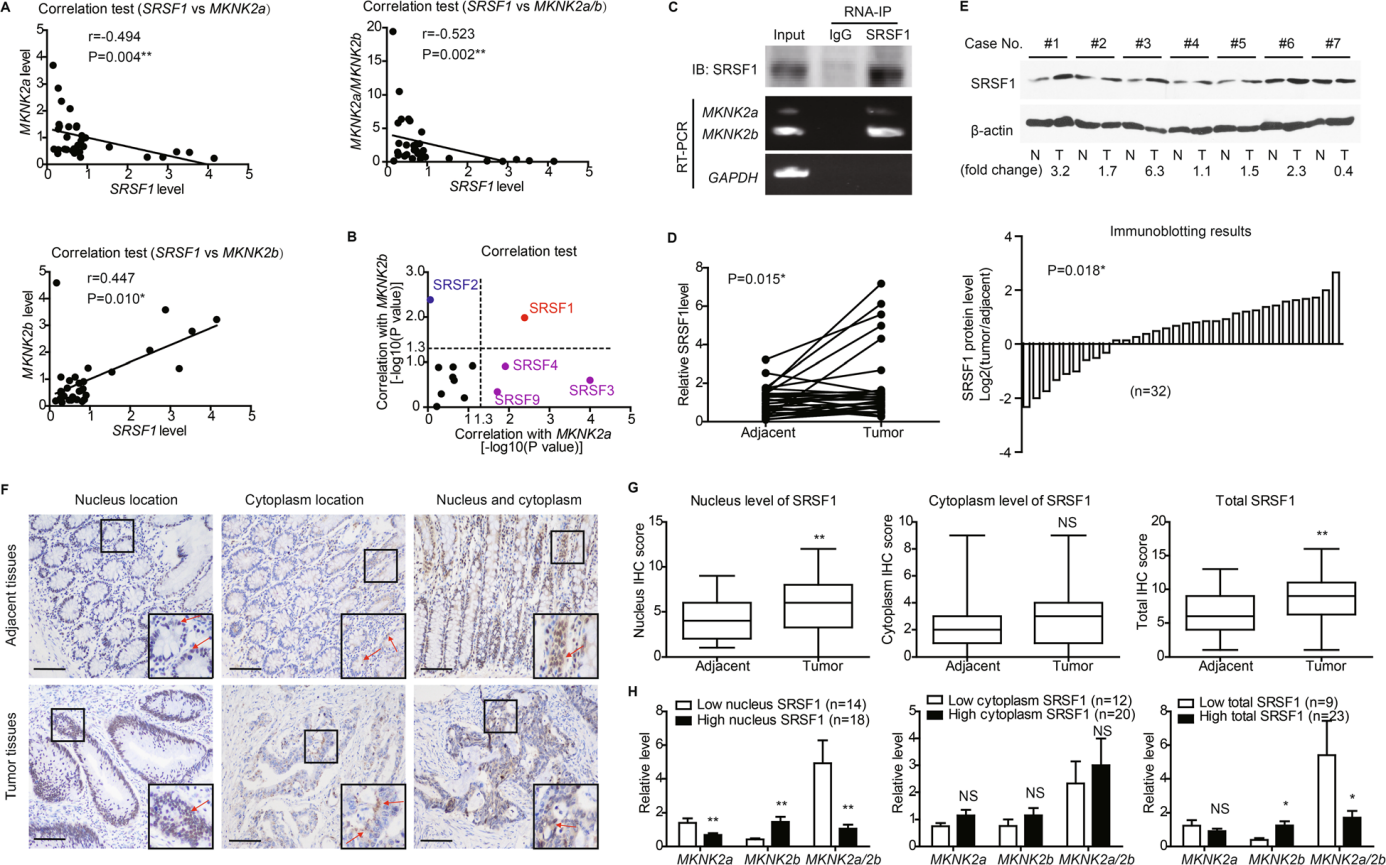
Correlation between nucleus SRSF1 location and *MKNK2* alternative splicing. **a** *SRSF1* level was tested by RT-qPCR in clinical CAC tissues (*n* = 32), and its correlations with *MKNK2* variants were analyzed by Spearman correlation test. **b** RNA levels of other well-known splicing factors were also measured by RT-qPCR. Spearman correlation tests showed that only *SRSF1* was significantly correlated with both *MKNK2a* and *MKNK2b* levels, indicating its potential effect on *MKNK2a*-*MKNK2b* switch. **c** SW480 cells transfected with pcDNA-vector or SRSF1 constructs were subjected to RNA-IP using anti-SRSF1 antibody in formaldehyde crosslinked nuclear extracts. The existence of SRSF1 protein and *MKNK2*-mRNA were tested via Western blotting and RT-PCR, respectively. **d** The mRNA levels of *SRSF1* in 32 paired CAC tissues and adjacent nontumorous tissues were compared by RT-qPCR. *P* value was based on paired Student’s t-test. **e** Protein levels of SRSF1 in clinical specimens were also tested by western blotting (upper panel), and 71.9% cases (23/32) showed increased SRSF1 in tumor tissues (lower panel). **f** Immunohistochemical staining identified different subcellular location of SRSF1 in tissue specimens, including nucleus location and cytoplasm location. Scale bar: 100 μm. **g** Differences of SRSF1 IHC scores between CAC and adjacent nontumorous tissues were represented by box plots (*n* = 32). Top and bottom box edges represented the third and first quartile. Whiskers indicated highest and lowest score. *P* value was based on paired Student’s t-test. **h** By classifying patients into different subgroups according to ROC curves (Fig. S3D), we found that tissues with higher nucleus SRSF1 level or total SRSF1 level expressed more *MKNK2b* variant and showed a lower *MKNK2a/2b* ratio. In contrast, no statistical difference was observed based on cytoplasm SRSF1 level. *P* value was calculated by unpaired Student’s t-test

*MKNK2* can be alternatively spliced to *MKNK2a* and *MKNK2b* mRNAs and consequently translated into two protein isoforms: Mnk2a and Mnk2b (Fig. 1a). By analyzing the mRNAs level of *MKNK2* isoforms in 32 paired tissues (Table S1), we found that *MKNK2a* was downregulated in CAC tumor tissues while *MKNK2b* was upregulated (Fig. 1b). Mutations of *KRAS* and *TP53* are the most well-characterized onco-driver of CAC, and we next investigated whether they have effects on *MKNK2* alternative splicing (Fig. 2c). Although *TP53* seemed to have no impact on *MKNK2* mRNA levels, *MKNK2a* splicing was inhibited while *MKNK2b* was remarkably upregulated in *KRAS*-mutated CAC samples. Therefore, we enrolled both *KRAS*-WT cells (Caco-2 and HT-29) and *KRAS*-mutated cells (HCT-116 and SW480) to conduct cellular validation. Comparing with Caco-2 and HT-29 cells, HCT-116 and SW480 cells showed lower *MKNK2a* but higher *MKNK2b* mRNA levels (Fig. 1d). Furthermore, transfecting Caco-2/HT-29 cells with *KRAS*-G12V plasmids significantly altered the predominant *MKNK2* isoform from *MKNK2a* to *MKNK2b* (Fig. 1e), indicating that *KRAS* can affect *MKNK2* alternative splicing. We next aimed to seek whether the *KRAS*-G12V-induced *MKNK2b* was resulted from increased RNA synthesis or decreased RNA degradation using NCM460 cells. Actinomycin D is a potent inhibitor that can inhibit poly(A)RNA synthesis in a dose-dependent manner [16]. According to our data, actinomycin D treatment significantly attenuated the *KRAS*-G12V-induced *MKNK2b* expression at 20 nM or higher concentrations (Fig. 1f), implying this *MKNK2b* alteration was correlated with RNA synthesis instead of RNA degradation. Interestingly, *MKNK2a* and *MKNK2b* showed opposite effects on *KRAS*-G12V-induced colony formation of NCM460 cells (Fig. 1g), indicating their distinct roles in modulating tumor development.

### *MKNK2* alternative splicing is correlated with tumor growth instead of LN metastasis

Additionally, *MKNK2a* was relatively lower in tumors with larger size while *MKNK2b* was higher in larger CAC tissues (Fig. 1h). Spearman correlation test showed that tumor diameter was negatively correlated to *MKNK2a* level and positively correlated to *MKNK2b* level (Fig. S1A, S1B). Apparently, the *MKNK2a*/*MKNK2b* ratio was decreased in tumor specimens with larger diameter (Fig. S1C). In contrast, we didn’t identify any significant correlation between *MKNK2* mRNA levels with lymph node (LN) metastasis (Figure S1D), indicating *MKNK2* may have little effect on modulating tumor invasion. Since we showed that *MKNK2* alternative splicing is closely correlated with *KRAS* mutation, we chose HCT-116 and SW480 cell for following cellular tests in this study. Although transfection of Mnk2a had no obvious effect on CAC cell proliferation (Fig. 1i) nor colony formation (Fig. 1j), overexpressing Mnk2b significantly enhanced cell proliferation capacity. Both our clinical and cellular findings suggested a novel tumor-promoting role of Mnk2b on CAC progression.

### Nucleus SRSF1 is closely correlated with *MKNK2* alternative splicing in tumor tissues

Since *KRAS* is not a splicing factor and is less possible for directly regulating *MKNK2* alternative splicing, we next aimed to figure out the upstream splicing factors targeting *MKNK2*. Serine/arginine-rich splicing factors (SRSFs) are the major splicing factors that regulate alternative splicing process in mammalian cells [17]. By testing the mRNA levels of *SRSF* family members, we found that *SRSF2* showed a positive relationship with *MKNK2b* level. In contrast, *SRSF3*, *SRSF4* and *SRSF9* showed negative correlations with *MKNK2a* level (Figure S2, Table S2). However, only *SRSF1* was negatively correlated with *MKNK2a* and positively correlated with *MKNK2b* simultaneously, indicating its predominant role on modulating *MKNK2a*-*MKNK2b* switch in CAC tissues (Fig. 2a, b). Moreover, RNA-immunoprecipitation results confirmed the interaction between SRSF1 protein and *MKNK2a/2b*-mRNA in SW480 cells (Fig. 2c).

Therefore, we further investigated whether SRSF1 participate in CAC progression via *MKNK2a*-*MKNK2b* switch. RT-qPCR and immunoblotting data showed that SRSF1 was upregulated in tumor tissues compared with adjacent nontumorous tissues on both RNA and protein levels (Fig. 2d, e). Interestingly, IHC data identified a diffused staining of SRSF1 protein in both nucleus and cytoplasm (Fig. 2f). To better evaluate the alteration of SRSF1 in CAC tissues, we separately assessed its expression in nucleus or cytoplasm. No statistical difference was observed between tumor tissues and adjacent tissues regarding to cytoplasm-SRSF1. Nevertheless, CAC samples showed higher total SRSF1 and nucleus-SRSF1 than nontumorous tissues (Fig. 2g). In addition, both total SRSF1 and nucleus-SRSF1 were positively correlated to *MKNK2b*-mRNA level, while negatively correlated to *MKNK2a* and *MKNK2a*/*MKNK2b* (Figure S3A, S3B). In contrast, cytoplasm-SRSF1 showed no significant correlation with *MKNK2* alternative splicing (Figure S3C). Using ROC method (Figure S3D), we next divided the primary cohort into subgroups based on SRSF1 levels in different subcellular locations. Consistent with Spearman correlation test, Chi-square results also revealed a significant difference of *MKNK2* alternative splicing between low nucleus-SRSF1 and high nucleus-SRSF1 groups (Fig. 2h). Similar results were obtained based on total SRSF1 expression but not cytoplasm-SRSF1 (Fig. 2h).

### TNPO3-dependent nucleus transportation of SRSF1 determines *MKNK2* alternative splicing and cell proliferation

**Fig. 3:**
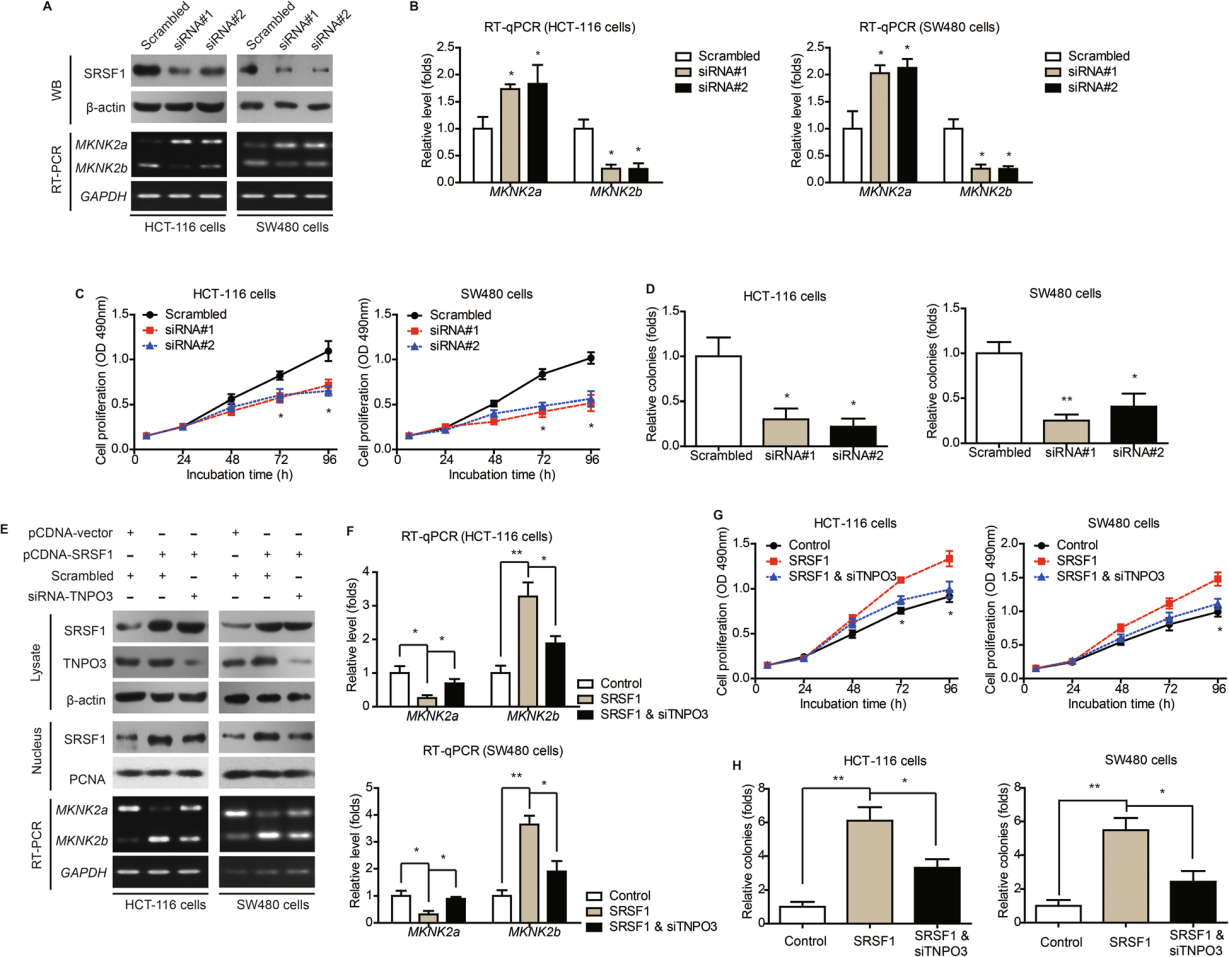
Nucleus transportation of SRSF1 exerts oncogenic effect via upregulating *MKNK2b* level. **a** HCT-116 and SW480 cells were transfected with either scrambled siRNA or siRNAs targeting SRSF1. The transfection efficiencies were verified by western blotting. The alterations of *MKNK2* alternative splicing were tested by RT-PCR. **b** Quantitative PCR results further confirmed the role of SRSF1 on regulating *MKNK2* alternative splicing. *P* value was based on unpaired Student’s t-test comparing with scrambled group. **c** MTT and colony formation (**d**) assays demonstrated that SRSF1-knockdown inhibited CAC cell proliferation. *P* value was based on unpaired Student’s t-test comparing with scrambled group. **e** Western blot results showed that silencing TNPO3 downregulated nucleus SRSF1 level, indicating nucleus importing of SRSF1 was at least partially depend on TNPO3. Moreover, RT-PCR data indicated that silencing TNPO3 attenuated the effect of SRSF1 on altering *MKNK2* alternative splicing. **f** Quantitative PCR results further verified the role of TNPO3-siRNA on attenuating SRSF1-induced *MKNK2a-MKNK2b* switch. *P* value was based on unpaired Student’s t-test comparing with SRSF1 group. **g** MTT and colony formation (**h**) assays demonstrated that TNPO3-knockdown attenuated the oncogenic effect of SRSF1 on CAC cell proliferation. *P* value was based on unpaired Student’s t-test comparing with SRSF1 group

We set out to explore the possible effects of SRSF1 on *MKNK2* alternative splicing in CAC cells. Knockdown of SRSF1 can significantly inhibit *MKNK2a*-*MKNK2b* switch according to RT-PCR (Fig. 3a) and RT-qPCR data (Fig. 3b), respectively. Of note, cell proliferation and colony formation capacities were also attenuated by silencing SRSF1 (Fig. 3c, d). On the other hand, overexpressing SRSF1 resulted in *MKNK2a*-*MKNK2b* switch and enhanced cell proliferation (Fig. 3e-h). Taking into consideration that only nucleus-SRSF1, instead of cytoplasm-SRSF1, showed a significant correlation with *MKNK2* alternative splicing in CAC samples, we hypothesize that nucleus transportation is critical for SRSF1-guided *MKNK2a*/*MKNK2b* switch. Previous studies had revealed that transportin 3 (TNPO3) can assist nucleus trafficking of SRSF members [18], thus we blocked the nucleus transportation of SRSF1 by silencing TNPO3 (Figure S3E). Accordingly, TNPO3-siRNA abolished the role of SRSF1 on promoting *MKNK2a*-*MKNK2b* switch (Fig. 3e, f) and resulted in attenuated cell proliferation (Fig. 3g, h).

### SRPK1 and SRPK2 are positively correlated with *MKNK2a*-*MKNK2b* switch and SRSF1 nucleus translocation

**Fig. 4:**
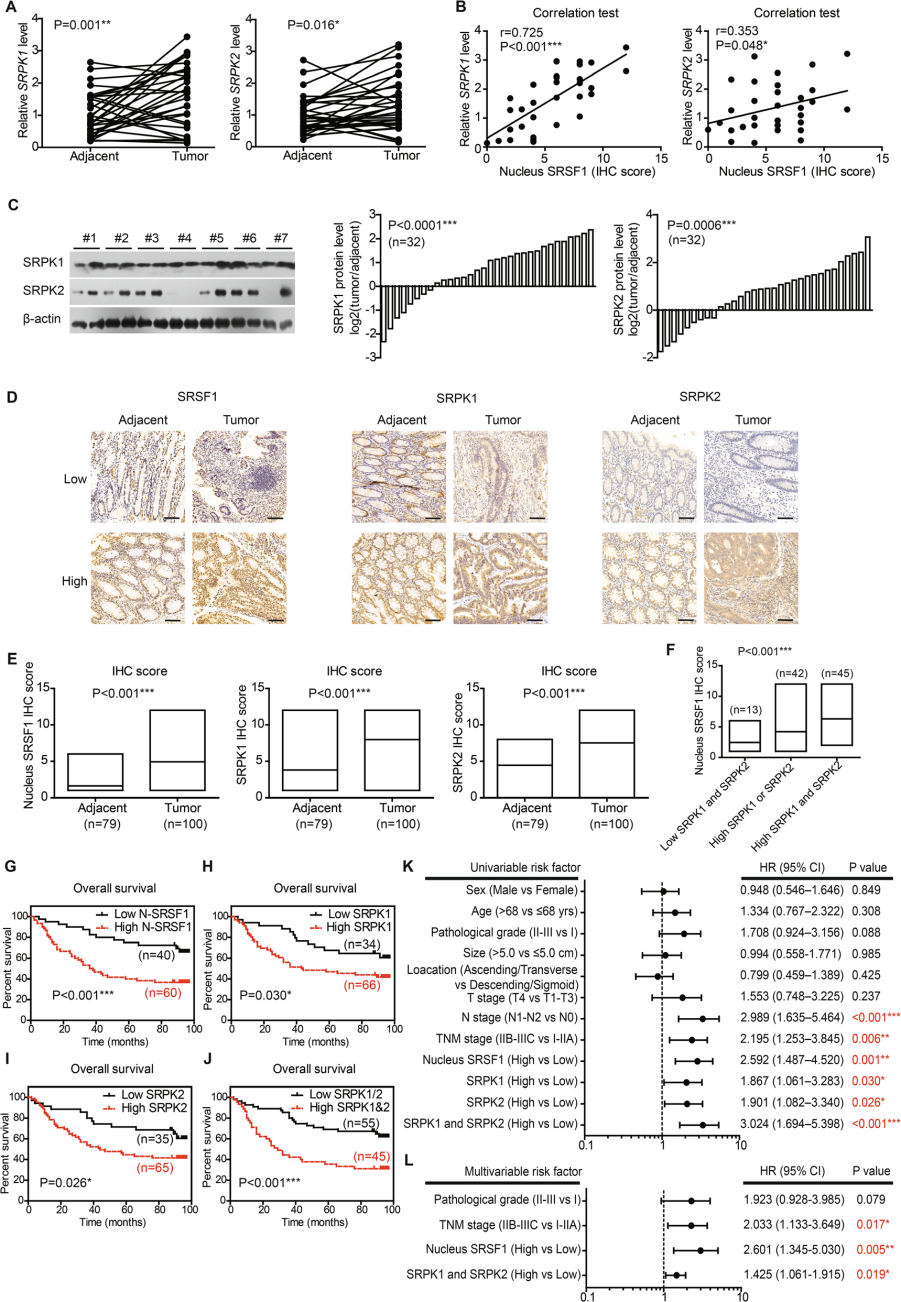
Expression and prognostic role of SRPKs in CAC. **a** RNA levels of upstream kinases targeting splicing factors in paired clinical specimens were tested by RT-qPCR (*n* = 32). P value was based on paired Student’s t-test. **b** Correlations between nucleus SRSF1 level and upstream kinases were analyzed via Spearman correlation test (*n* = 32). The nucleus SRSF1 level was scored by the specific nucleus staining of SRSF1 (ranging 0–12) without considering its cytosol staining. **c** Protein levels of SRPK1 and SRPK2 in paired clinical specimens were tested by western blotting (left panel). After semi-quantified, we found that 75% cases (24/32) showed increased SRPK1 in tumor tissues (middle panel), and 71.9% cases (23/32) exhibited increased SRPK2 (right panel). **d** Representative IHC results of SRSF1, SRPK1, and SRPK2 in specimens from validation cohort. Scale bar: 100 μm. **e** The different protein levels of SRPK1, SPRK2, and nucleus SRSF1 in validation cohort were exhibited by box plots according to IHC data. *P* value was based on unpaired Student’s t-test. **f** Patients with both high-SRPK1 and high-SRPK2 levels exhibited the highest nucleus SRSF1 level, while patients with low SRPK1 and low SRPK2 showed the lowest nucleus SRSF1 IHC score. *P* value was based on One-way ANOVA test. Kaplan-Meier survival curves showed the clinical relevance of nucleus SRSF1 (**g**), SRPK1 (**h**), and SRPK2 (**i**) in validation cohort of CAC patients, respectively. Furthermore, the higher expression of both SRPK1 and SRPK2 showed a more significant role on indicating poorer overall survival (**j**). Forest plots summarized the significant prognostic factors by univariate (**k**) and multivariate (**l**) analyses, the corresponding data were supplemented in Table S4 and Table S5. *P* value was calculated by log-rank test or Cox-regression test

Since phosphorylation plays vital roles on intracellular trafficking of SRSF proteins [19], we were interested to identify the upstream kinases responsible for SRSF1 nucleus transportation. CDC2-like kinase 1 (CLK1) and SR-specific protein kinases (SRPKs) had been proved to phosphorylate the RS domain of SRSF1 through in vitro kinase assays [20]. Therefore, we tested the statistical relevance between *MKNK2a*-*MKNK2b* switch and *CLK1, SRPK1, SRPK2*, respectively. Correlation test revealed that *CLK1* has no significant correlation with *MKNK2* alternative splicing (Fig. S4A). In contrast, the level of *SRPK1* mRNA is positively correlated with *MKNK2b* level, while negatively correlated with *MKNK2a* level or *MKNK2a*/*MKNK2b* ratio (Figure S4B). As for *SRPK2*, although no significance was observed towards *MKNK2a* level, it showed a significant correlation with *MKNK2b* (Fig. S4C). Besides, both *SRPK1* and *SRPK2* mRNA levels were upregulated in tumor tissues comparing with adjacent tissues in our primary cohort (Fig. 4a). TCGA database also revealed upregulated transcriptions of *SRSF1* and *SRPK*s (Figure S5). Moreover, *SRPK1* as well as *SRPK2* showed positive correlations with nucleus-SRSF1 protein level (Fig 4b), indicating that SRPKs may promote nucleus translocation of SRSF1. We next evaluated protein expression level of SRPKs in primary cohort by immunoblotting. Consistent with mRNA data, both SRPK1 and SRPK2 proteins were elevated in tumor tissues (Fig. 4c).

### Prognostic effects of SRSF1, SRPK1, and SRPK2

The findings in primary cohort and TCGA database promoted us to further investigate the prognostic significance of SRSF1 and SRPKs by introducing another independent validation cohort (*n* = 100). IHC experiments were conducted to explore the protein expression and localization. SRPK1 and SRPK2 were predominantly localized in cytoplasm, while SRSF1 was stained in both nucleus and cytoplasm (Fig. 4d). In accordance with primary cohort, CAC samples in validation cohort showed higher nucleus localization of SRSF1 and higher expression of SRPK1/2 (Fig. 4e). Besides, patients with higher SRPK1 or SRPK2 were characterized with higher nucleus-SRSF1 level (Fig. 4f). Furthermore, higher SRPK1 was observed in patients with larger tumor size and advanced tumor stages (Table S3).

Kaplan-Meier survival analyses revealed that patients with higher nucleus-SRSF1 had a poorer overall survival (Fig. 4g). Higher SRPK1 or higher SRPK2 also indicated unfavorable prognosis, respectively (Fig. 4h, i). Of note, patients with both high SRPK1 and high SRPK2 showed a lowest overall survival time comparing with other subgroups (Fig. 4j). Prognostic effects of other clinicopathological factors were also summarized, which demonstrated the clinical significance of lymph node metastasis and TNM stage (Fig. 4k, Table S4). Additionally, multivariate analysis was performed to explore independent prognostic variables (Fig. 4l, Table S5). Higher nucleus-SRSF1, higher SRPK1/2, and advanced TNM stage were all identified as independent risk factors of CAC overall survival.

### Restoration of Mnk2b reverses the anti-proliferation effects of SRPK knockdown

**Fig. 5:**
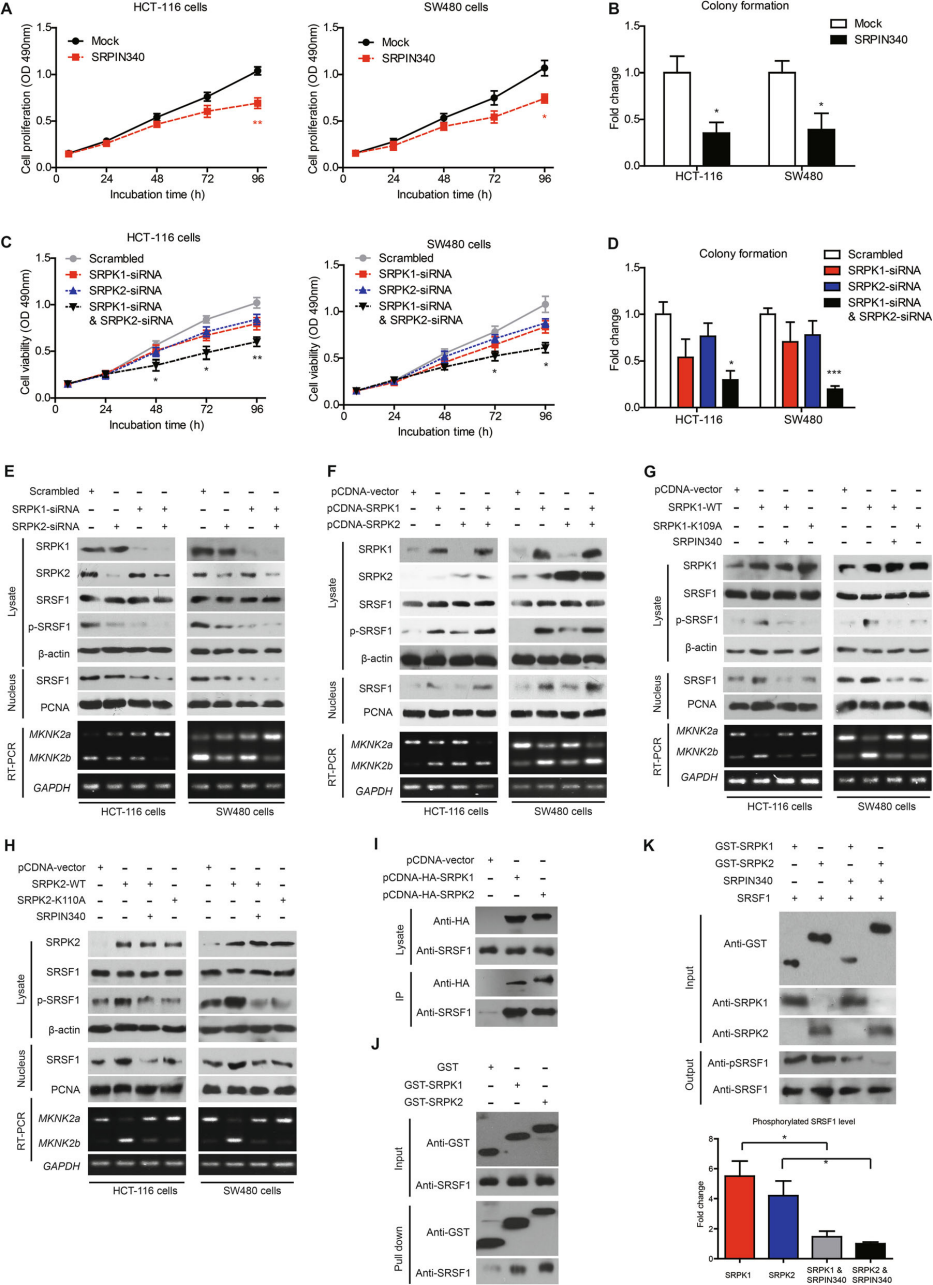
SRPKs promote SRSF1 phosphorylation, nucleus transportation, and downstream *MKNK2* alternative splicing. **a** Treatment with SRPKs inhibitor, SRPIN340, resulted in decreased proliferation of HCT-116 and SW480 cells. *P* value was based on unpaired Student’s t-test comparing with mock group which was treated with DMSO. **b** Colony formation assay also demonstrated an anti-proliferation effect of SRPIN340 on CAC cells, indicating the oncogenic role of SRPKs. *P* value was based on unpaired Student’s t-test comparing with mock group. **c** MTT and colony formation (**d**) assays demonstrated that silencing either SRPK1 or SRPK2 may impair CAC proliferation, although the statistical difference was not significant. Knockdown of both SRPK1 and SRPK2 can significantly inhibit CAC cell proliferation. *P* value was based on unpaired Student’s t-test comparing with scrambled group. **e** Knockdown of either SRPK1 or SRPK2 attenuated phosphorylation of SRSF1 (p-SRSF1) and reduced nucleus SRSF1 (N-SRSF1) level. RT-PCR results showed a positive correlation between *MKNK2b* and SRPKs. Furthermore, simultaneously knockdown of SRPK1 and SRPK2 exhibited the most significant effects. Semi-quantification of immunoblotting results and corresponding RT-qPCR data were shown in Supplementary Figure S7A-B. **f** Overexpressing SRPKs increased phosphorylation of SRSF1 and promoted its nucleus translocation according to immunoblotting results. Semi-quantification of immunoblotting results and corresponding RT-qPCR data were shown in Supplementary Figure S7C-D. **g** SRPIN340 treatment towards SRPK1-overexpressing cells abolished the effects above. Consistently, the kinase dead (KD) mutant SRPK1-K109A, lost effects on modulating SRSF1 phosphorylation, subcellular location, and *MKNK2a-MKNK2b* switch. Semi-quantification of immunoblotting results and corresponding RT-qPCR data were shown in Supplementary Figure S7E-F. **h** SRPIN340 treatment towards SRPK2-overexpressing cells also abolished its effects on modulating SRSF1 phosphorylation, subcellular location, and *MKNK2a-MKNK2b* switch. The KD mutant of SRPK2, namely SRPK2-K110A, showed similar effects with SRPIN340. Semi-quantification of immunoblotting results and corresponding RT-qPCR data were shown in Supplementary Figure S7G-H. **i** SW480 cells were transfected with HA-tagged SRPK1 (pcDNA-SRPK1) or HA-tagged SRPK2 (pcDNA-SRPK2) or pcDNA-vector constructs. After lysing cells and conducting immunoprecipitation assay with anti-HA agarose, we found that SRPK1 and SRPK2 can interact with SRSF1 in CAC cells. **j** Purified GST-tagged SRPK1 or GST-SRPK2 proteins were incubated with purified SRSF1 proteins in vitro using GST protein as negative control. GST pull-down assay demonstrated the direct interaction between SRPK1/2 with SRSF1. The input was shown in upper panel. **k** The kinase activity of SRPK1/2 targeting SRSF1 was tested by in vitro kinase assay. Purified SRSF1 was incubated with GST-SRPK1 or GST-SRPK2 with/without SRPIN340 (10 μM) as described in the Method section. Reaction products were separated via SDS-PAGE and analyzed by western blotting. Phosphorylation of SRSF1 in reaction products was semi-quantified after normalized by total SRSF1 (lower panel). The data showed that both SRPK1 and SRPK2 can directly phosphorylate SRSF1, which can be inhibited by the inhibitor SRPIN340

The effect of SRPKs on CAC progression were validated by cellular experiments. The nonspecific SRPK inhibitor, SRPIN340, can significantly inhibit cell proliferation and colony formation of CAC cells (Fig. 5a, b). After testing the transfection efficiency of SRPK1-siRNAs and SRPK2-siRNAs (Figure S6A, S6B), we knocked down the two kinases in CAC cells and found that silencing either SRPK1 or SRPK2 showed no statistically significant effect on cell proliferation. However, simultaneously knockdown of both SRPK1 and SRPK2 remarkably attenuated cell proliferation and colony formation (Fig. 5c, d). Interestingly, overexpressing Mnk2b restored the cell proliferation capacity of SRPK1/2-silenced cells (Figure S6C-E). In contrast, SRPK1 and SRPK2 overexpression enhanced cell proliferation while TNPO3-siRNA almost abolished this effect (Figure S6F-H).

### SRPK1 and SRPK2 regulate *MKNK2* alternative splicing by directly phosphorylating SRSF1 and promoting SRSF1 nucleus transportation

We also compared the effects of SRPK1/2 knockdown or SRPK1/2 overexpression on SRSF1 phosphorylation, nucleus shuttling, and *MKNK2* alternative splicing in CAC cell lines (Fig. 5e, f; Figure S7A-D). As expected, either SRPK1-siRNA or SRPK2-siRNA significantly inhibited SRSF1 phosphorylation and nucleus translocation. In contrast, SRPK1 or SRPK2 overexpression upregulated the phosphorylation and nucleus accumulation of SRSF1. Besides, overexpressing SRPK1/2 led to an enhanced *MKNK2a*-*MKNK2b* switch, while silencing SRPK1/2 showed opposite effects according to RT-PCR and RT-qPCR results. To further investigate whether kinase activity of SRPK1/2 were critical for SRSF1-guided *MKNK2* alternative splicing, we generated the kinase-dead mutants SRPK1-K109A and SRPK2-K110A. Either kinase-dead mutant or inhibitor SRPIN340 significantly attenuated, if not abolished, the role of SRPK1/2 on modulating SRSF1 phosphorylation, nucleus transportation as well as downstream *MKNK2* alternative splicing (Fig. 5g, h; Figure S7E-H). Moreover, immunoprecipitation data identified the intracellular interaction between SRSF1 with SRPK1/2 (Fig. 5i). Extracellular GST pull down assay was then conducted and confirmed their direct binding activity (Fig. 5j). We next investigate whether the interaction between SRPK1/2 and SRSF1 is a kinase-substrate process. In vitro phosphorylation test showed that both SRPK1 and SRPK2 are capable of phosphorylating SRSF1, which can be inhibited by SRPIN340 (Fig. 5k). Therefore, SRPK1 and SRPK2 may directly phosphorylate SRSF1 and promote it nucleus translocation, subsequently modulate *MKNK2* alternative splicing.

### PP1α activity is downregulated in colon adenocarcinoma tissues

**Fig. 6:**
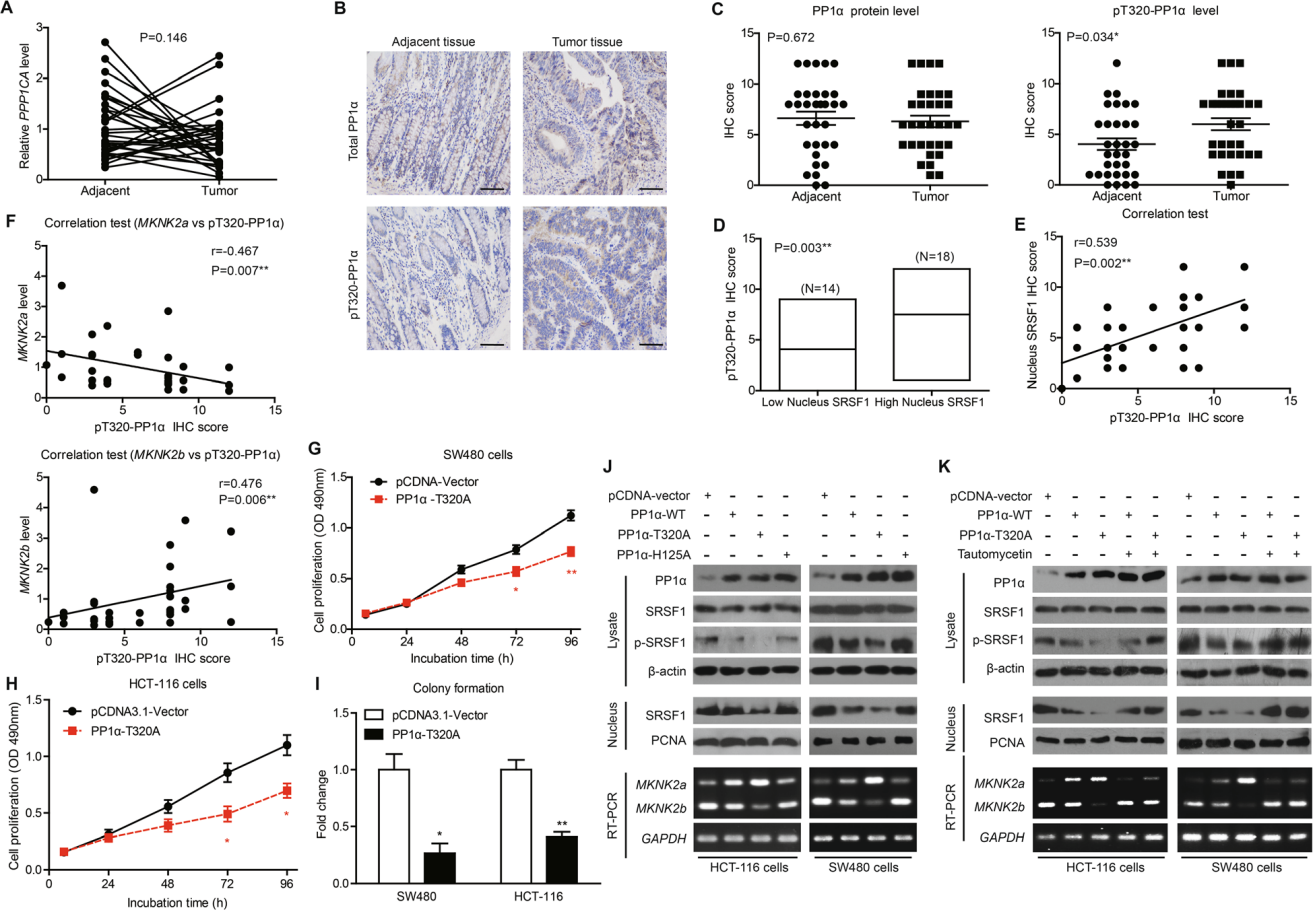
PP1α activity is decreased in CAC tissues and correlates with SRSF1-dependent *MKNK2* alternative splicing. **a** There was no statistical difference of *PPP1CA* mRNA levels between tumor tissues and adjacent nontumorous tissues. *P* value was based on paired Student’s t-test (*n* = 32). **b** Representative IHC images showed the immunoreactivities of total PP1α and phosphorylated-PP1α (pT320-PP1α) in clinical specimens. Scale bar: 100 μm. **c** Although no difference regarding total PP1α protein level was observed between tumor and adjacent tissues (left panel), tumor tissues exhibited higher phosphorylation on T320 site (pT320-PP1α, right panel) based on IHC data. *P* value was based on paired Student’s t-test (*n* = 32). **d** Tumor tissues with higher nucleus SRSF1 showed higher pT320-PP1α levels. P value was based on unpaired Student’s t-test. **e** Spearman correlation test further confirmed a positive correlation between nucleus SRSF1 and pT320-PP1α levels. **f** Spearman correlation test identified a negative correlation between pT320-PP1α and *MKNK2a* levels. In contrast, *MKNK2b* was positively correlated with pT320-PP1α level. **g** Proliferation capacity of SW480 cells or HCT-116 cells (**h**) was impaired by overexpressing pcDNA-PP1α-T320A, a constitutively active PP1α construct, as revealed by MTT assays. *P* value was based on unpaired Student’s t-test. **i** Colony formation assay was conducted for the cells described in (**g**) and (**h**), further indicating that PP1α-T320A may attenuate CAC proliferation. **j** CAC cells were transfected with three different PP1α constructs, including PP1α-WT, PP1α-T320A (constitutively active mutant), and PP1α-H125A (kinase-dead mutant) using pcDNA-vector as control. The levels of phosphorylated SRSF1, nucleus SRSF1 were measured by western blotting, and the *MKNK2* splicing were evaluated by RT-PCR (lower panel). Semi-quantification of immunoblotting results and corresponding RT-qPCR data were shown in Supplementary Figure S8D. (K) CAC cells were transfected with PP1α-WT or PP1α-T320A with/without tautomycetin treatment. The levels of phosphorylated SRSF1, nucleus SRSF1 were measured by western blotting, and *MKNK2* splicing were evaluated by RT-PCR (lower panel). Semi-quantification of immunoblotting results and corresponding RT-qPCR data were shown in Supplementary Figure S8E

It has been reported that PP1α is the major phosphatase that dephosphorylates SRSF1 [21], thus we explored whether PP1α is responsible for antagonizing SRPK1/2 during CAC development. To our surprise, no significant difference of *PPP1CA* mRNA level was observed between tumor tissues and adjacent tissues (Fig. 6a). Taking into consideration that the phosphatase activity of PP1α is as important as its expression level, we next tested whether the activity of PP1α was altered in CAC tissues by testing its phosphorylation on T320 site, which inhibits its catalyzation capacity. IHC data showed cytoplasmic localization of both PP1α and pT320-PP1α (Fig. 6b). Consistent with RT-qPCR data, PP1α protein level showed no difference between tumor and adjacent tissues. However, the pT320-PP1α level was upregulated in tumor tissues, indicating a decreased PP1α activity (Fig. 6c).

### PP1α negatively modulates SRSF1 nucleus transportation, *MKNK2* alternative splicing, and tumor proliferation

Furthermore, patients with higher nucleus-SRSF1 showed higher pT320-PP1α level but not total PP1α level (Fig. 6d; Figure S8A). Spearman correlation test also revealed a positive correlation between pT320-PP1α and nucleus-SRSF1 (Fig. 6e) while no correlation was observed regarding total PP1α (Figure S8B). Similarly, although no association was identified between total PP1α and *MKNK2* alternative splicing (Figure S8C), pT320-PP1α was positively correlated to *MKNK2a*-*MKNK2b* switch (Fig. 6f).

In addition, the constitutively active mutant of PP1α, PP1α-T320A, significantly inhibited proliferation of CAC cells (Fig. 6g-i). Cellular assays were then performed to test crosstalk between PP1α and SRSF1 by overexpressing constitutively active mutant (T320A) or inactive mutant (H125A) of PP1α, respectively. We found that either PP1α-WT or PP1α-T320A can decrease SRSF1 phosphorylation and nucleus translocation, while PP1α-H125A showed opposite effects (Fig. 6j; Figure S8D). Consistently, PP1α-T320A inhibited *MKNK2a*-*MKNK2b* switch while PP1α-H125A promoted it (Fig. 6j; Figure S8D). The PP1α specific inhibitor, tautomycetin, can efficiently block the effects of PP1α-WT or PP1α -T320A on altering SRSF1 phosphorylation, nucleus translocation and *MKNK2* alternative splicing (Fig. 6k; Figure S8E). Furthermore, we observed similar findings on SW620 cells, the metastatic cell line of SW480 (Figure S9). Therefore, we concluded that PP1α can suppress CAC progression at least partially by inhibiting SRSF1-guided *MKNK2a*-*MKNK2b* switch, which is dependent on SRSF1 phosphorylation and nucleus shuttling.

### SRPKs and PP1α have opposite effects on modulating *MKNK2* alternative splicing and tumor growth in mice model

**Fig. 7:**
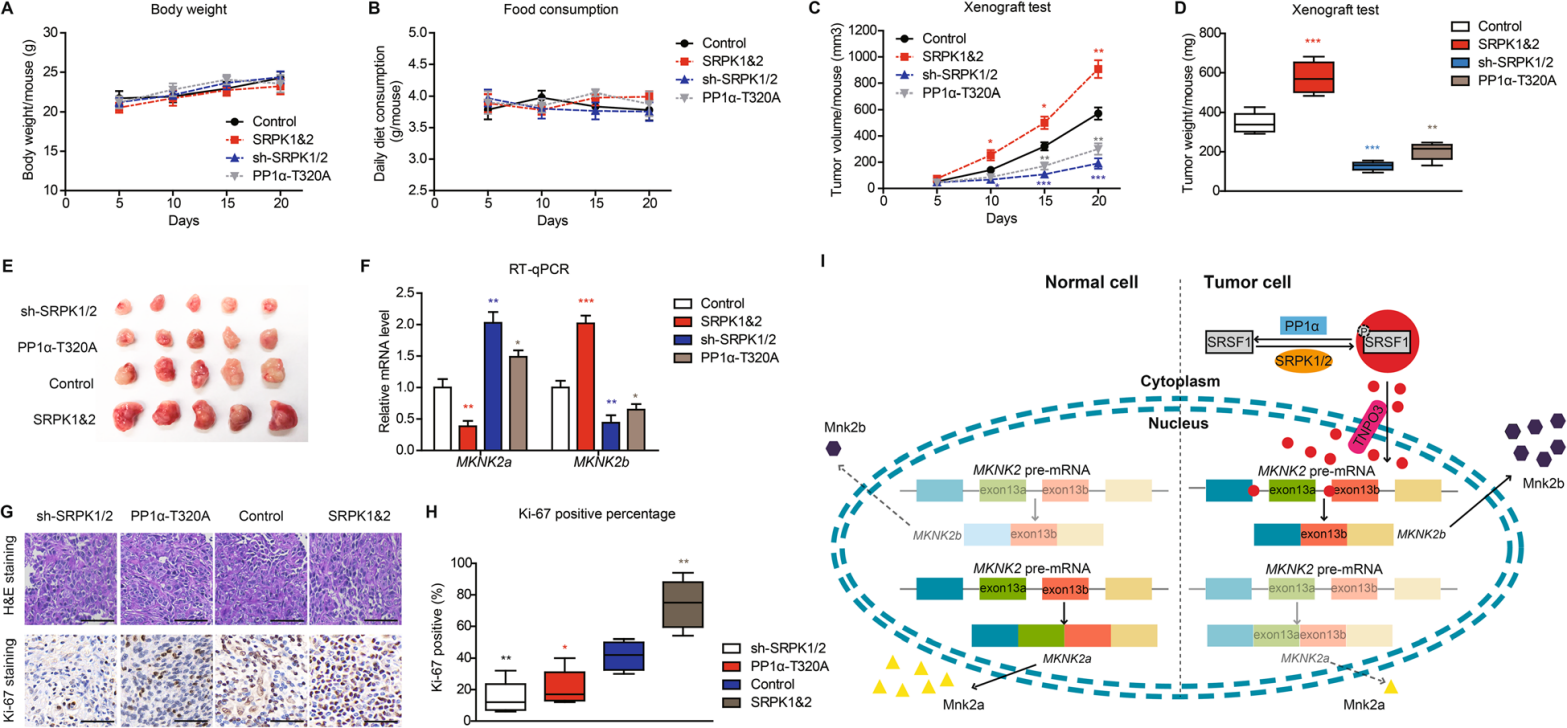
SRPKs and PP1α have opposite effects on *MKNK2* alternative splicing and tumor growth in mice model. SW480 cells transduced with shSRPK1/2, or overexpressing SRPK1&2, or overexpressing PP1α-T320A were subcutaneously injected into six-week male Balb/c nude mice. The mice condition was monitored from the date of observing macroscopical xenograft. The body weight (**a**) or food consumption (**b**) of mice in each group showed no statistical difference. **c** Xenograft growth curves were plotted as described in the Method section. The mice were scarified after monitoring for 20 days, then the xenografts were isolated, weighted (**d**), and photographed (**e**). **f** Levels of *MKNK2a* and *MKNK2b* splicing variants were tested via RT-qPCR from isolated xenografts. **g** Representative H&E staining and Ki-67 immunostaining results of isolated xenografts. Scale bar: 100 μm. **h** The proliferation index was determined using the percentage of Ki-67 positive cells in isolated xenografts. **i** A schematic model shows the effects of SRPK1/2 and PP1α on regulating *MKNK2a/MKNK2b* switch. *MKNK2* pre-mRNA can be alternatively spliced into two *MKNK2* mRNA isoforms and further translated into two protein isoforms, namely Mnk2a and Mnk2b. Mnk2a perhaps play an anti-tumor role while Mnk2b exerts remarkable oncogenic effects according to our data. In nontumorous cells, Mnk2a is the predominantly protein isoform compared to Mnk2b. In colon adenocarcinoma cells, SRPK1 and SRPK2 are upregulated while PP1α is inactivated, resulting into a hyperphosphorylation status of SRSF1. Phosphorylation of SRSF1 promotes its nucleus transportation in a TNPO3-dependent manner. The nucleus SRSF1 can directly bind to *MKNK2* pre-mRNA and significantly generate more *MKNK2b* isoform, while the *MKNK2a* proportion is decreased

Xenograft models were utilized to verify tumor-related roles of SRPK1/2 and PP1α in vivo. SW480 cells that stably overexpressed SRPK1&2 or PP1α, or stably silenced SRPK1/2 with shRNAs were seeded subcutaneously in Balb/c nude mice. There was no statistical difference among the groups on the aspects of body weight nor food consumption (Fig. 7a, b). Among the four groups, SRPK1&2-overexpressing group showed the highest proliferation capacity and largest tumor size. In contrast, PP1α-T320A group and sh-SRPK1/2 group both showed impaired growth process comparing to control group (Fig. 7b-e).

The mRNA levels of *MKNK2a* and *MKNK2b* in isolated xenografts were further tested by RT-qPCR, which revealed consistent results with cellular assays. On one hand, SRPK1&2 downregulated *MKNK2a* splicing and upregulated *MKNK2b* level. On the other hand, PP1α-T320A or sh-SRPK1/2 attenuated *MKNK2a*-*MKNK2b* switch (Fig. 7f). Proliferation index of xenografts was also evaluated by calculating Ki-67 positive staining (Fig. 7g). As a result, SRPK1&2 group showed the highest Ki-67 staining percentage, while PP1α-T320A group and sh-SRPK1/2 group showed decreased Ki-67 level, which is consistent with tumor growth curve (Fig. 7h). Taken together, our data revealed an oncogenic role of SRPK1/2 signaling in colon adenocarcinoma cells (Fig. 7i). In nontumorous cell, *MKNK2* pre-mRNA is predominantly spliced into *MKNK2a* and translated to Mnk2a protein, which exerts anti-tumor effects. In colon adenocarcinoma cells, SRSF1 is hyperphosphorylated due to elevated SRPK1/2 and decreased PP1α activity, resulting in SRSF1 nucleus shuttling. Nucleus SRSF1 accumulation finally leads to alterative splicing of *MKNK2* pre-mRNA to *MKNK2b* and upregulated the Mnk2b oncoprotein level (Fig. 7i).

## Discussion

Alternative splicing expands the proteomic complexity by generating splicing variants on mRNA level and encoded distinct protein isoforms including kinases. In the present study, we focused on *MKNK2a* and *MKNK2b* alternative splicing, encoding Mnk2a and Mnk2b, respectively. Our data revealed a downregulation of *MKNK2a* and an upregulation of *MKNK2b* in colon adenocarcinomas, which is closely correlated with *KRAS* mutation. The hyper-synthesis of *MKNK2b* is observed in samples with larger tumor size, implying its role on promoting tumor proliferation. Maimon et al. had recently reported that Mnk2b is deficient on activating p38-induced cell death pathway, while maintaining its capacity of promoting eIF4E-induced oncoprotein translation. In contrast, Mnk2a can induce both oncogenesis as well as p38-MAPK stress apoptosis pathway. Therefore, they elucidated downstream mechanism of how Mnk2a and Mnk2b exert opposite effects on tumor progression in Ras-activated cells [10]. Consistently, our data also revealed a significant correlation between *MKNK2* splicing shift with *KRAS* mutation in colon adenocarcinomas, while no obvious association with *TP53* was identified. Additionally, cell lines possessing *KRAS* mutation showed a prevalence of *MKNK2a*-*MKNK2b* switch in comparison with *KRAS*-WT cell lines. Strikingly, *KRAS*-G12V transfection enhanced *MKNK2b* mRNA splicing even in nontumorous colon epithelial cells, indicating an oncogenesis correlation between *KRAS* mutation and *MKNK2* alternative splicing. According to the data reported by Maimon et al. [10], oncogenic transformation caused by activated *Ras* can induce *MKNK2a-MKNK2b* switch in breast cancer cells, pancreatic cancer cells and in lung cancer cells. Furthermore, coexpressing Mnk2a inhibited the oncogenic phenotype of *Ras*-transformed MCF-10A cells, indicating that Mnk2a can block *Ras*-induced transformation [10]. Therefore, it is high likely that oncogenic *Ras* induces tumor transformation at least partially through affecting *MKNK2a-MKNK2b* switch. Another study by Maimon et al. artificially induced a strong switch from *MKNK2b* to *MKNK2a* by using designated oligonucleotides, resulting in inhibited glioblastoma development and re-sensitization to chemotherapy [22]. Their study provides evidence on the therapeutic potential of manipulating *MKNK2* alternative splicing as a novel approach to treat glioma. Our present results regarding *MKNK2a*-*MKNK2b* switch in colon adenocarcinoma implicated that similar intervention may be a novel direction for CAC treatment.

In addition, our RT-qPCR screening assay indicated SRSF1 as a promising modulator targeting *MKNK2* alternative splicing, which was confirmed by correlation test, RNA immunoprecipitation, and knockdown assays. The findings on that nuclear SRSF1, instead of cytoplastic SRSF1, was correlated with patients’ clinical outcomes promoted us to further explore the upstream regulation mechanisms of SRSF1 subcellular trafficking. Consistent with electron microscopy data [23], we verified the critical role of TNPO3 on importing SRSF1 into nucleus of colon adenocarcinoma cells. Further study showed that the tumor-promoting role of SRSF1 can be significantly attenuated by silencing TNPO3, confirming that only nucleus SRSF1 exerts pro-oncogenic effects.

Post-translational modification has remained the focus of protein function investigation during the past decades, and phosphorylation is one of the most important modifications which we focused on [24, 25]. Abnormal expression of kinases or phosphatases can result in dysregulated phosphorylation events, leading to various malignancies as we previously reported [14, 26]. It has been reported that multisite phosphorylation of SRSF1 is a prerequisite for its nucleus transportation [27, 28], which is the key mechanism for its functional alternative splicing. Therefore, we next aimed to investigate the upstream kinase and phosphatase which controls SRSF1 phosphorylation. Three major kinases had been reported to phosphorylate SRSF1 including SRPK1, SRPK2, and CLK1 [19, 20]. Considering that enzyme-substrate relationship can be difference in various cell types, we initially explored whether those kinases could affect SRSF1 nucleus translocation in colon adenocarcinoma cells. Although we didn’t observe any statistical correlation between CLK1 and nucleus SRSF1 level, both SRPK1 and SRPK2 indeed correlated with SRSF1 nucleus translocation. Additionally, SRPK1 and SRPK2 showed upregulated expression in CAC tissues compared with adjacent tissues, which is consistent with their expression patterns in acute myeloid leukemia, breast cancer, and pancreatic cancer [29–31].

In accordance with their underlying crosstalk, SRPK1/2 and SRSF1 are all identified as independent risk factors for the overall survival of CAC patients. Following cellular results again confirmed that silencing SRPK1 and SRPK2 can lead to decreased SRSF1 phosphorylation, nucleus transportation, *MKNK2a*-*MKNK2b* switch, and cell proliferation. Similar results were obtained by introducing kinase-dead mutants or using their inhibitor SRPIN340. Of note, we systematically verified that both SRPK1 and SRPK2 can directly interact and phosphorylate SRSF1 by immunoprecipitation, GST pull-down, and in vitro phosphorylation test. The anti-tumor effects of SRPK inhibition had been recently reported in other malignancies [31, 32], our data enlarged the therapeutic potential of developing SRPK inhibitor for CAC treatment. Interestingly, a previous study by Wang et al. demonstrated that SRPK1 can act as both oncogene and tumor suppressor, either higher or lower level of SRPK1 induces cell transformation [33]. The underlying mechanism is determined by the interaction between SRPK1 and PHLPP, thus aberrant SRPK1 expression in either direction could result in hyperactivation of Akt by blocking its phosphatase PHLPP. Considering that specific effect of kinase is largely depend on its substrate, identification of other SRPK1 substrates will be invaluable for further illustrating its tumor-related role. Anyway, here our data confirmed the pro-oncogenic axis of SRPK1/2-SRSF1-*MKNK2* in CAC progression.

Besides kinases, we next focused on SRSF1 upstream phosphatase to impartially evaluate the importance of its phosphorylation in CAC. Adams JA et.al and her colleagues reported that protein phosphatase 1α (PP1α) directly dephosphorylates the RS domain of SRSF1 through binding to its RNA recognition motif 1 (RRM1) through an allosteric mechanism [34]. Of note, disrupting the interaction between PP1α and SRSF1 results in significant shifts of alternative splicing such as EIF5 and TIMM8B genes [35]. On the other hand, PP1α has been demonstrated to inhibit the metastasis of SW620 colon cancer cells via deactivating Src protein [36]. Therefore, we were promoted to assess whether PP1α was dysregulated in colon adenocarcinoma. Although we didn’t find any significant difference of PP1α expression between CAC tissues and adjacent tissues, its catalytic inactivate type, namely the Tyr320-phosphorylated PP1α, was elevated in tumor tissues, indicating that PP1α activity was downregulated in colon adenocarcinoma. Here we also validated the role of PP1α on regulating *MKNK2* alternative splicing by introducing its constitutively active mutant and inactive mutant, respectively. Besides SW480 and HCT-116 cells, PP1α and its mutations showed similar effects on the metastatic SW620 cell line, further highlighting the prognostic value of PP1α-SRSF1-MNK2 axis in CAC progression. However, we have to keep in mind that phosphorylation status of SR proteins can not only modulate their subcellular localization but also determines the splicing site selection [37], thus the more detailed effects of SRPKs and PP1α on alternative splicing need further validation. Together with the findings from xenografts, we concluded that PP1α can attenuates colon cancer progression at least partially via antagonizing SRPK1/2-induced SRSF1 phosphorylation and downstream *MKNK2a*-*MKNK2b* splicing shift.

## Conclusions

Our study reveals a significant role of SRSF1-guided *MKNK2a*-*MKNK2b* splicing switch in the proliferation of colon adenocarcinoma, which is determined by SRSF1 phosphorylation and subcellular translocation. The alternative splicing of *MKNK2* is balanced by SRPK1/2 and PP1α, thus providing opportunities of therapeutic intervention, such as SRPK inhibitors or PP1α allosteric activators, in treating malignancies. Additionally, the subcellular location of SRSF1, expression levels of SRPK1/2, as well as phosphorylation status of PP1α can all serve as independent prognostic predictors for the overall survival of colon adenocarcinoma.

## Supplementary Information

**Additional file 1: Table S1.** Patients information of primary cohort. **Table S2.** Correlations between *MKNK2* alternative splicing and splicing factors on mRNA levels. **Table S3**. Correlation between protein expression level and clinical features of colon adenocarcinoma patients in validation cohort. **Table S4**. Kaplan-Meier survival analyses for colon adenocarcinoma patients. **Table S5.** Cox-regression analysis for overall survival of colon adenocarcinoma patients. **Table S6.** Antibodies, chemicals, siRNAs, shRNAs, and primers in this study. **Figure S1.** Correlations between MKNK2 alternative splicing with patients’ characteristics in primary cohort. **Figure S2.** Correlations between splicing factors and MKNK2 alternative splicing in CAC tissues. **Figure S3.** Correlations between SRSF1 protein expression and MKNK2 alternative splicing in CAC tissues. **Figure S4.** Correlations between upstream kinases and MKNK2 alternative splicing in CAC tissues. **Figure S5.** SRSF1, SRPK1, and SRPK2 are upregulated in CAC tissues. **Figure S6.** Effects of SRPKs on cell proliferation can be altered by Mnk2b and TNPO3. **Figure S7.** SRPKs modulate SRSF1 phosphorylation, nucleus transportation, and MKNK2 alternative splicing. **Figure S8.** Functions of PP1α on modulating SRSF1-dependent MKNK2 splicing. **Figure S9.** Functions of PP1α on modulating proliferation and SRSF1-dependent MKNK2 splicing in metastatic SW620 cells.

## Data Availability

The data regarding the current study are available on reasonable request.

## Abbreviations

CAC: Colon adenocarcinoma
CLK1: CDC2-like kinase 1
eIF4E: Eukaryotic translation initiation factor 4E
Mnk: MAP kinase-interacting serine/threonine-protein kinase
PP1α: Protein phosphatase 1α
RNA-IP: RNA immunoprecipitation
SRSF: Serine and arginine rich splicing factor
SRPK: SR-specific protein kinase
TNPO3: Transportin 3
