# Supplementary material for: SRPK1/2 and PP1α exert opposite functions by modulating SRSF1-guided MKNK2 alternative splicing in colon adenocarcinoma
Source: J Exp Clin Cancer Res. 2021 Feb 18;40:75. doi: 10.1186/s13046-021-01877-y (PMC7893936; doi:10.1186/s13046-021-01877-y)
Supplement: Supplementary file 1 — Additional file 1: Table S1. Patients information of primary cohort. Table S2. Correlations between MKNK2 alternative splicing and splicing factors on mRNA levels. Table S3. Correlation between protein expression level and clinical features of colon adenocarcinoma patients in validation cohort. Table S4. Kaplan-Meier survival analyses for colon adenocarcinoma patients. Table S5. Cox-regression analysis for overall survival of colon adenocarcinoma patients. Table S6. Antibodies, chemicals, siRNAs, shRNAs, and primers in this study. Figure S1. Correlations between MKNK2 alternative splicing with patients’ characteristics in primary cohort. Figure S2. Correlations between splicing factors and MKNK2 alternative splicing in CAC tissues. Figure S3. Correlations between SRSF1 protein expression and MKNK2 alternative splicing in CAC tissues. Figure S4. Correlations between upstream kinases and MKNK2 alternative splicing in CAC tissues. Figure S5. SRSF1, SRPK1, and SRPK2 are upregulated in CAC tissues. Figure S6. Effects of SRPKs on cell proliferation can be altered by Mnk2b and TNPO3. Figure S7. SRPKs modulate SRSF1 phosphorylation, nucleus transportation, and MKNK2 alternative splicing. Figure S8. Functions of PP1α on modulating SRSF1-dependent MKNK2 splicing. Figure S9. Functions of PP1α on modulating proliferation and SRSF1-dependent MKNK2 splicing in metastatic SW620 cells. [file 13046_2021_1877_MOESM1_ESM.docx]

**Table S1.** Patients information of primary cohort.

| **No.** | **Gender** | **Age** | **Location** | **Differentiation** | **Size (cm)** | **T stage** | **Lymph node** | **TNM** | ***KRAS*** |
| --- | --- | --- | --- | --- | --- | --- | --- | --- | --- |
| 1 | Male | 60 | Descending | Moderate | 8.7 | III | Positive | IIIb | Wild type |
| 2 | Male | 66 | Transverse | Well | 4.1 | II | Positive | IIIa | Wild type |
| 3 | Female | 47 | Sigmoid | Moderate | 9.2 | IV | Negative | IIb | Wild type |
| 4 | Male | 74 | Sigmoid | Well | 6.3 | II | Negative | I | G12V |
| 5 | Male | 59 | Sigmoid | Moderate | 5.1 | III | Negative | IIa | Wild type |
| 6 | Male | 68 | Ascending | Moderate | 4.8 | III | Positive | IIIb | G13D |
| 7 | Male | 62 | Sigmoid | Well | 3.3 | II | Negative | I | Wild type |
| 8 | Male | 60 | Sigmoid | Moderate | 9.5 | III | Positive | IIIb | Wild type |
| 9 | Female | 72 | Transverse | Well | 6.9 | III | Positive | IIIb | G13D |
| 10 | Male | 64 | Ascending | Moderate | 6.0 | II | Positive | IIIa | G12D |
| 11 | Female | 66 | Sigmoid | Moderate | 3.8 | III | Negative | IIa | Wild type |
| 12 | Male | 81 | Sigmoid | Well | 10.0 | IV | Negative | IIb | Wild type |
| 13 | Female | 79 | Ascending | Moderate | 4.7 | III | Positive | IIIb | Wild type |
| 14 | Male | 64 | Ascending | Moderate | 5.6 | III | Negative | IIa | Wild type |
| 15 | Male | 72 | Ascending | Poor | 8.3 | III | Negative | IIa | Wild type |
| 16 | Male | 59 | Descending | Moderate | 4.8 | III | Negative | IIa | Q61L |
| 17 | Male | 55 | Sigmoid | Moderate | 10.0 | IV | Negative | IIb | G13D |
| 18 | Male | 55 | Sigmoid | Moderate | 2.9 | I | Negative | I | Wild type |
| 19 | Female | 72 | Ascending | Well | 5.9 | II | Negative | I | Wild type |
| 20 | Male | 52 | Sigmoid | Moderate | 6.6 | IV | Positive | IIIc | Wild type |
| 21 | Female | 74 | Descending | Moderate | 8.0 | IV | Negative | IIb | Wild type |
| 22 | Female | 70 | Ascending | Moderate | 8.0 | III | Negative | IIa | G12D |
| 23 | Male | 75 | Sigmoid | Moderate | 6.5 | III | Positive | IIIb | Wild type |
| 24 | Female | 57 | Transverse | Moderate | 10.5 | III | Positive | IIIb | G12V |
| 25 | Male | 61 | Descending | Well | 9.3 | III | Negative | IIa | Wild type |
| 26 | Female | 48 | Sigmoid | Well | 7.7 | III | Negative | IIa | G13D, A14V |
| 27 | Male | 68 | Ascending | Well | 1.8 | II | Negative | I | G12A |
| 28 | Female | 56 | Ascending | Moderate | 2.6 | III | Positive | IIIb | Wild type |
| 29 | Female | 70 | Ascending | Well | 4.2 | II | Negative | I | Wild type |
| 30 | Female | 59 | Ascending | Well | 4.5 | III | Negative | IIa | G12D |
| 31 | Female | 63 | Sigmoid | Poor | 9.8 | III | Positive | IIIb | G12V |
| 32 | Male | 57 | Sigmoid | Well | 2.4 | II | Positive | IIIa | Wild type |

|  | *MKNK2a* | | *MKNK2b* | | *MKNK2a/2b* | |
| --- | --- | --- | --- | --- | --- | --- |
|  | r | P value | r | P value | r | P value |
| *SRSF1* | -0.4938 | 0.0041** | 0.4471 | 0.0103* | -0.5227 | 0.0021** |
| *SRSF2* | 0.03026 | 0.8694 | 0.4934 | 0.0041** | -0.2892 | 0.1084 |
| *SRSF3* | -0.654 | <0.0001*** | 0.2093 | 0.2504 | -0.4471 | 0.0103* |
| *SRSF4* | -0.438 | 0.0122* | 0.2782 | 0.1231 | -0.4193 | 0.0169* |
| *SRSF5* | -0.134 | 0.4647 | 0.1221 | 0.5055 | -0.1752 | 0.3375 |
| *SRSF6* | -0.112 | 0.5416 | 0.2734 | 0.13 | -0.1976 | 0.2783 |
| *SRSF7* | -0.3155 | 0.0786 | 0.2802 | 0.1204 | -0.3468 | 0.0518 |
| *SRSF8* | -0.0962 | 0.6003 | 0.00852 | 0.9631 | -0.022 | 0.9049 |
| *SRSF9* | -0.4115 | 0.0193* | 0.1372 | 0.4541 | -0.2474 | 0.1721 |
| *SRSF10* | -0.2211 | 0.224 | 0.2085 | 0.2522 | -0.2375 | 0.1905 |
| *SRSF11* | -0.2124 | 0.2431 | 0.2255 | 0.2145 | -0.2639 | 0.1444 |
| *SRSF12* | -0.2768 | 0.1251 | 0.09114 | 0.6198 | -0.1811 | 0.3212 |
| *HNRNPA1* | -0.2149 | 0.2375 | 0.2755 | 0.1269 | -0.2679 | 0.1382 |

**Table S2.** Correlations between *MKNK2* alternative splicing and splicing factors on mRNA levels.

* P<0.05, ** P<0.01, *** P<0.001 by Spearman correlation test.

**Table S3**. Correlation between protein expression level and clinical features of colon adenocarcinoma patients in validation cohort.

| **Variables** | **Patients** | **Nucleus SRSF1 level** | | **P value** | **SRPK1 level** | | **P value** | **SRPK2 level** | | **P value** |
| --- | --- | --- | --- | --- | --- | --- | --- | --- | --- | --- |
|  | **(n=100)** | **Low (n=40)** | **High (n=60)** |  | **Low (n=34)** | **High (n=66)** |  | **Low (n=35)** | **High (n=65)** |  |
| **Sex** |  |  |  | 0.414 |  |  | 0.673 |  |  | 0.834 |
| Female | 50 | 22 | 28 |  | 16 | 34 |  | 18 | 32 |  |
| Male | 50 | 18 | 32 |  | 18 | 32 |  | 17 | 33 |  |
| **Age (years)** |  |  |  | 0.514 |  |  | 0.571 |  |  | 0.950 |
| ≤ 68 | 51 | 22 | 29 |  | 16 | 35 |  | 18 | 33 |  |
| > 68 | 49 | 18 | 31 |  | 18 | 31 |  | 17 | 32 |  |
| **Pathological grade** |  |  |  | 0.356 |  |  | 0.516 |  |  | 0.487 |
| Grade I | 23 | 12 | 11 |  | 6 | 17 |  | 10 | 13 |  |
| Grade II | 61 | 23 | 38 |  | 21 | 40 |  | 21 | 40 |  |
| Grade III | 16 | 5 | 11 |  | 7 | 9 |  | 4 | 12 |  |
| **Size (largest diameter)** |  |  |  | 0.010* |  |  | 0.009** |  |  | 0.912 |
| ≤ 5.0 cm | 65 | 32 | 33 |  | 28 | 37 |  | 23 | 42 |  |
| > 5.0 cm | 35 | 8 | 27 |  | 6 | 29 |  | 12 | 23 |  |
| **Location** |  |  |  | 0.142 |  |  | 0.261 |  |  | 0.721 |
| Ascending/Transverse | 49 | 16 | 33 |  | 14 | 35 |  | 18 | 31 |  |
| Descending/Sigmoid | 51 | 24 | 27 |  | 20 | 31 |  | 17 | 34 |  |
| **T stage** |  |  |  | 0.046* |  |  | 0.327 |  |  | 0.707 |
| T1-T2 | 6 | 5 | 1 |  | 3 | 3 |  | 3 | 3 |  |
| T3 | 75 | 30 | 45 |  | 27 | 48 |  | 26 | 49 |  |
| T4 | 19 | 5 | 14 |  | 4 | 15 |  | 6 | 13 |  |
| **N stage** |  |  |  | 0.132 |  |  | 0.007** |  |  | 0.117 |
| N0 | 61 | 28 | 33 |  | 27 | 34 |  | 25 | 36 |  |
| N1-N2 | 39 | 12 | 27 |  | 7 | 32 |  | 10 | 29 |  |
| **TNM stage** |  |  |  | 0.142 |  |  | 0.001** |  |  | 0.367 |
| Stage I-IIA | 51 | 24 | 27 |  | 25 | 26 |  | 20 | 31 |  |
| Stage IIB-IIIC | 49 | 16 | 33 |  | 9 | 40 |  | 15 | 34 |  |

* P<0.05, ** P<0.01 by Chi-square test.

**Table S4**. Kaplan-Meier survival analyses for colon adenocarcinoma patients.

| **Variable** | **Cases**  **(n)** | **5-year OS (%)** | **Survival months**  **(Mean ± S.D.)** | **HR (95% CI)** | **P value** |
| --- | --- | --- | --- | --- | --- |
| **Sex** |  |  |  | 0.948 (0.546–1.646) | 0.849 |
| Female | 50 | 56.0% | 61.92 ± 5.32 |  |  |
| Male | 50 | 52.0% | 62.21 ± 5.30 |  |  |
| **Age (years)** |  |  |  | 1.334 (0.767–2.322) | 0.308 |
| ≤ 68 | 51 | 56.9% | 65.53 ± 4.95 |  |  |
| > 68 | 49 | 51.0% | 58.48 ± 5.63 |  |  |
| **Pathological grade** |  |  |  | 1.708 (0.924–3.156) | 0.088 |
| Grade I | 23 | 73.9% | 78.65 ± 5.57 |  |  |
| Grade II-III | 77 | 48.1% | 57.11 ± 4.43 |  |  |
| **Size (largest diameter)** |  |  |  | 0.994 (0.558–1.771) | 0.985 |
| ≤ 5.0 cm | 65 | 55.4% | 62.27 ± 4.71 |  |  |
| > 5.0 cm | 35 | 51.4% | 61.69 ± 6.22 |  |  |
| **Location** |  |  |  | 0.799 (0.459–1.389) | 0.425 |
| Ascending/Transverse | 49 | 49.0% | 58.91 ± 5.35 |  |  |
| Descending/Sigmoid | 51 | 58.8% | 65.11 ± 5.24 |  |  |
| **T stage** |  |  |  | 1.553 (0.748–3.225) | 0.237 |
| T1-T3 | 81 | 56.8% | 64.12 ± 4.17 |  |  |
| T4 | 19 | 42.1% | 53.32 ± 8.34 |  |  |
| **LN metastasis** |  |  |  | 2.989 (1.635–5.464) | <0.001*** |
| Negative | 61 | 63.9% | 72.32 ± 4.22 |  |  |
| Positive | 39 | 38.5% | 46.04 ± 6.19 |  |  |
| **TNM stage** |  |  |  | 2.195 (1.253–3.845) | 0.006** |
| Stage I-IIA | 51 | 64.7% | 72.38 ± 4.67 |  |  |
| Stage IIB-IIIC | 49 | 42.9% | 51.35 ± 5.52 |  |  |
| **Nucleus SRSF1 level** |  |  |  | 2.592 (1.487–4.520) | 0.001** |
| Low | 40 | 75.0% | 79.16 ± 4.69 |  |  |
| High | 60 | 40.0% | 50.72 ± 4.90 |  |  |
| **SRPK1 level** |  |  |  | 1.867 (1.061–3.283) | 0.030* |
| Low | 34 | 67.6% | 75.26 ± 5.30 |  |  |
| High | 66 | 47.0% | 55.29 ± 4.78 |  |  |
| **SRPK2 level** |  |  |  | 1.901 (1.082–3.340) | 0.026* |
| Low | 35 | 71.4% | 75.35 ± 5.53 |  |  |
| High | 65 | 44.6% | 54.92 ± 4.72 |  |  |
| **SRPK1 and SRPK2** |  |  |  | 3.024 (1.694–5.398) | <0.001*** |
| Low SRPK1 or SRPK2 | 55 | 69.1% | 75.11 ± 4.37 |  |  |
| High SRPK1 and SRPK2 | 45 | 35.6% | 46.13 ± 5.56 |  |  |

* P<0.05, ** P<0.01, *** P<0.001 by log-rank test.

**Table S5.** Cox-regression analysis for overall survival of colon adenocarcinoma patients.

| **Variable** | **HR** | **95% CI** | **P value** |
| --- | --- | --- | --- |
| **Pathological grade**  (Grade II-III vs Grade I) | 1.923 | 0.928-3.985 | 0.079 |
| **TNM stage**  (Stage IIB-IIIC vs Stage I-IIA) | 2.033 | 1.133-3.649 | 0.017* |
| **Nucleus SRSF1**  (High vs Low) | 2.601 | 1.345-5.030 | 0.005** |
| **SRPK1 and SRPK2**  (High vs Low) | 1.425 | 1.061-1.915 | 0.019* |

Note: TNM stage involves N stage, therefore we didn’t enroll “*N stage*” as a parameter during Cox regression analysis to avoid bias. Similarly, we didn’t enroll “*SRPK1*” or “*SRPK2*” individually due to their correlation with variable “*SRPK1 and SRPK2*”.

* P<0.05, ** P<0.01 by Cox-regression test.

**Table S6.** Antibodies, chemicals, siRNAs, shRNAs, and primers in this study.

| **Antibodies** | **Cat.** | **Supplier** |
| --- | --- | --- |
| SRPK1 | sc-100443 | Santa Cruz |
| SRPK2 | sc-136078 | Santa Cruz |
| Mnk2 | sc-271559 | Santa Cruz |
| SRSF1 | sc-33652 | Santa Cruz |
| Phos-SRSF1 | MABE50 | Sigma-Aldrich |
| TNPO3 | sc-376346 | Santa Cruz |
| PP1α | 2582S | Cell Signaling Technology |
| PP1α-p-Thr320 | 2581S | Cell Signaling Technology |
| Ki-67 | sc-23900 | Santa Cruz |
| HA | sc-7392 | Santa Cruz |
| GST | sc-138 | Santa Cruz |
| β-actin | sc-47778 | Santa Cruz |
| PCNA | sc-56 | Santa Cruz |
|  |  |  |
| **Chemicals** | **Cat.** | **Supplier** |
| Actinomycin D | A1410 | Sigma-Aldrich |
| SRPIN340 | S7270 | Selleck Chemicals |
| Tautomycetin | 2305 | Tocris Bioscience |
|  |  |  |
| **siRNAs** | **Sequence** |  |
| SRSF1-siRNA#1 | ACGATTGCCGCATCTACGT |  |
| SRSF1-siRNA#2 | AGGACATTGAGGACGTG |  |
| TNPO3-siRNA#1 | CTGAATTACTGCCGTATTT |  |
| TNPO3-siRNA#2 | CGACATTGCAGCTCGTGTA |  |
| SRPK1-siRNA#1 | TTAATGACTTCAATCACTCCATTGC |  |
| SRPK1-siRNA#2 | TAAGAAATCTGTGAAGCCAGCTGCC |  |
| SRPK2-siRNA#1 | AATACTGCCTAGCAGCTCTATGATG |  |
| SRPK2-siRNA#2 | TCAGCTTGGTGATGTGTCGCAGTTC |  |
| Scramble-siRNA | TCGGCTCTTACGCATTCAA |  |
|  |  |  |
| **shRNAs** | **Sequence** |  |
| SRPK1-shRNA | CAAGAAGATCCTAATGATTA |  |
| SRPK2-shRNA | GCAGAGAGTGATTACACGTAT |  |
|  |  |  |
| **PCR primers** | **Forward** | **Reverse** |
| *MKNK2a* | GCTGCGACCTGTGGAGCCTGGG | GATGGGAGGGTCAGGCGTGGTC |
| *MKNK2b* | GCTGCGACCTGTGGAGCCTGGG | GAGGAGGAAGTGACTGTCCCAC |
| *GAPDH* | ATCAAGAAGGTGGTGAAGCAG | CTTACTCCTTGGAGGCCATGT |
|  |  |  |
| **QPCR primers** | **Forward** | **Reverse** |
| *MKNK2a* | TCCGTGACGCCAAGCAG | GGTCTTTGGCACAGCTG |
| *MKNK2b* | TCCGTGACGCCAAGCAG | GAGGAAGTGACTGTCCCAC |
| *SRPK1* | CCGAAAGAAAAGGACCAAGGCC | CCTGCTCTGGTAGATCACTGTC |
| *SRPK2* | CCTCGTTGTTCTCTGGATCCTTAGAACCTG | TCTATGGAGCGGTACTGACGCGTCTG |
| *PP1α* | TGTGGCGAGTTTGACAATGC | GGGCTTGAGGATCTGGAAAG |
| *SRSF1* | GAGTTCGAGGACCCGCGAGACG | GAGCTCCGCCACCTCCAC |
| *SRSF2* | CAAGTCCAGATCCGCACGAA | ACCATTTTCTTAAGAGGACACCG |
| *SRSF3* | AATTGGAACGGGCTTTTGGC | CCATCTAGCTCTCGGACTGC |
| *SRSF4* | CTAAGAGTAGATCTCGGTCCAGGT | GGGGCTCCTGCTTTTCTCT |
| *SRSF5* | GAGGCTTTGGTTTTGTGGAA | CGAGCCCTAGCATGTTCAAT |
| *SRSF6* | AAATACGGACCACCTGTTCG | CTTCACCTGCTTGTCGCATA |
| *SRSF7* | CGCTGGCAAAGGAGAGTTAG | CGAATTCCACAAAGGCAAAT |
| *SRSF8* | TCTGGGTCCTCCACTAGCTC | TCTTGGATCGCGACCTTGAC |
| *SRSF9* | TACGTGGGGAACCTTCCGA | GCATCCTCTGCATCTCGGGG |
| *SRSF10* | CGACAATGATAGACCAAACTGC | CCTTTGGTCGCTTGAACTGC |
| *SRSF11* | CAGTCCAGATGTCGTCAGCA | GGGTTCTCGCTCCTGTTGATT |
| *SRSF12* | TTGACTTCTACACTCGCCGC | ATTTGGCCTGGTGTTTTGCG |
| *HNRNPA1* | TGGATTTGGTAATGATGGAAGC | TCTCTGGCTCTCCTCTCCTG |
| *GAPDH* | TCACCACCATGGAGAAGGC | GCTAAGCAGTTGGTGGTGCA |


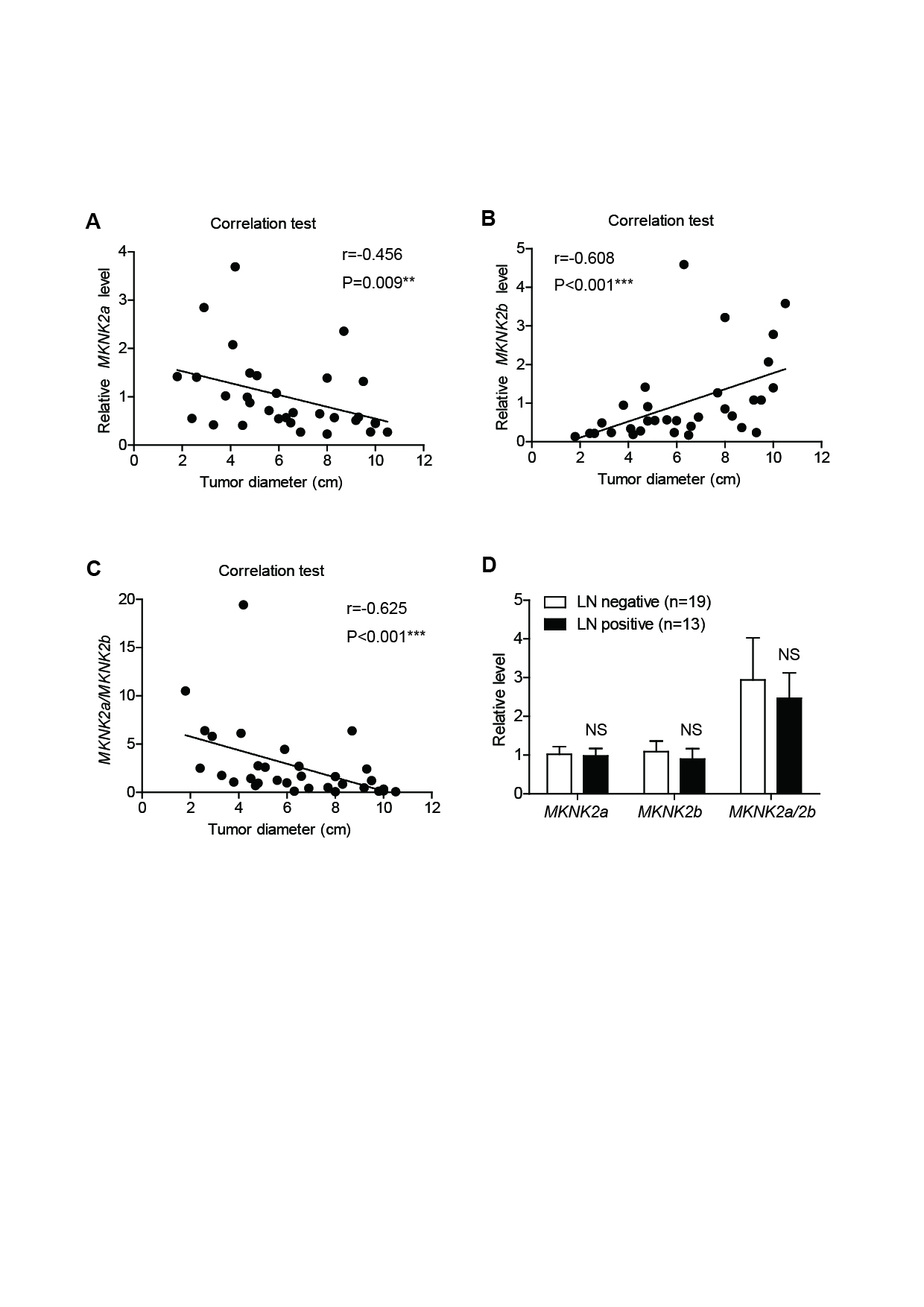


**Figure S1. Correlations between *MKNK2* alternative splicing with patients’ characteristics in primary cohort.**

Spearman correlation test was conducted to assess whether tumor size (n=32) was correlated with *MKNK2a* level (A), *MKNK2b* level (B), or *MKNK2a/2b* ratio (C). (D) There was no statistical difference between patients with or without lymph node metastasis regarding to *MKNK2* pre-mRNA splicing. P value was based on unpaired Student’s t-test.


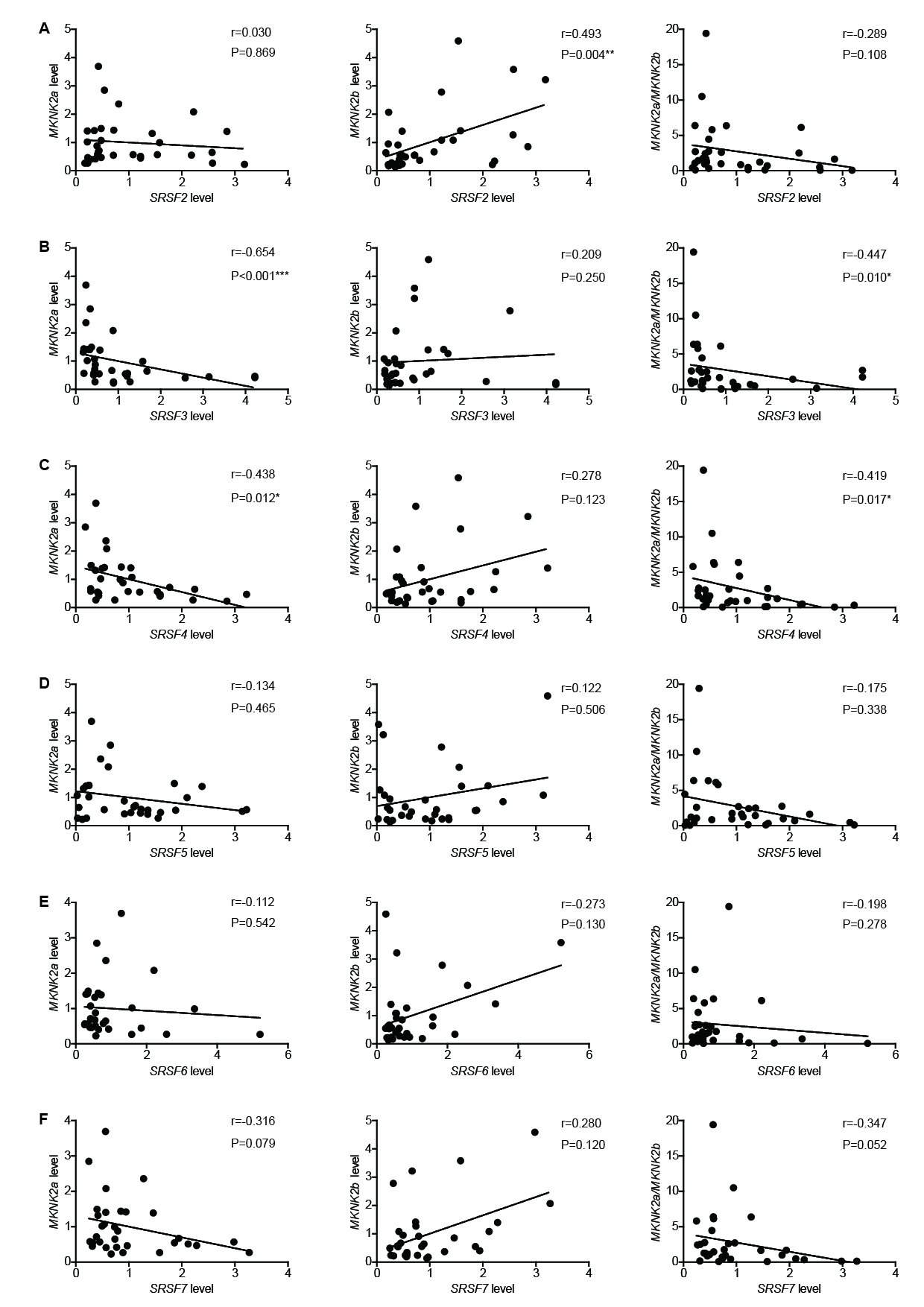


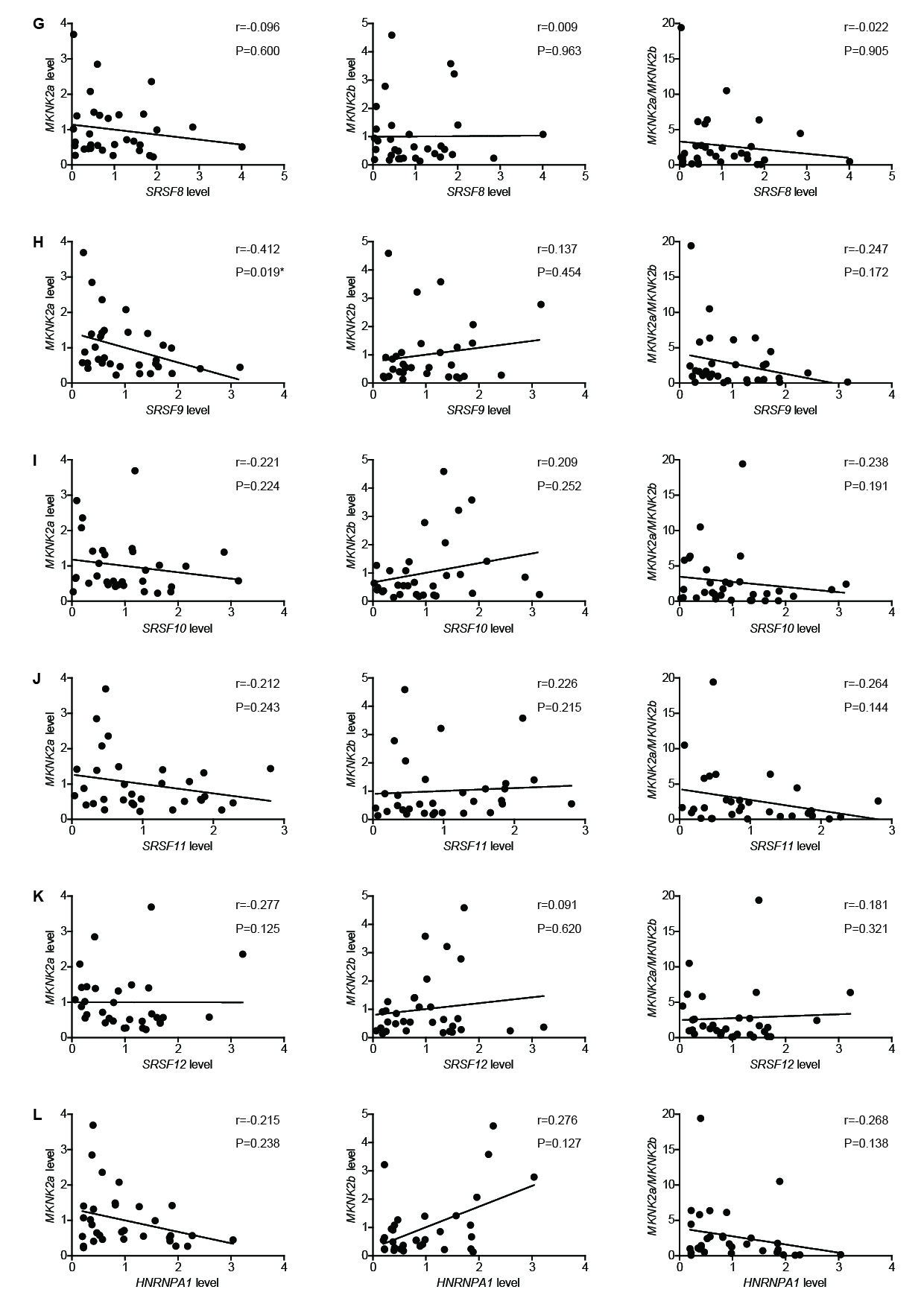
**Figure S2. Correlations between splicing factors and *MKNK2* alternative splicing in CAC tissues.**

The mRNA levels of well-known splicing factors were systematically screened by RT-qPCR, including *SRSF2, SRSF3, SRSF4, SRSF5, SRSF6, SRSF7, SRSF8, SRSF9, SRSF10, SRSF11, SRSF12*, and *HNRNPA1*. The non-SRSF member, HNRNPA1, was used as control. Their correlations with *MKNK2a, MKNK2b*, and *MKNK2a/2b* ratio were tested by Spearman correlation test (n=32), respectively. P values and correlation coefficient were summarized in Table S2.


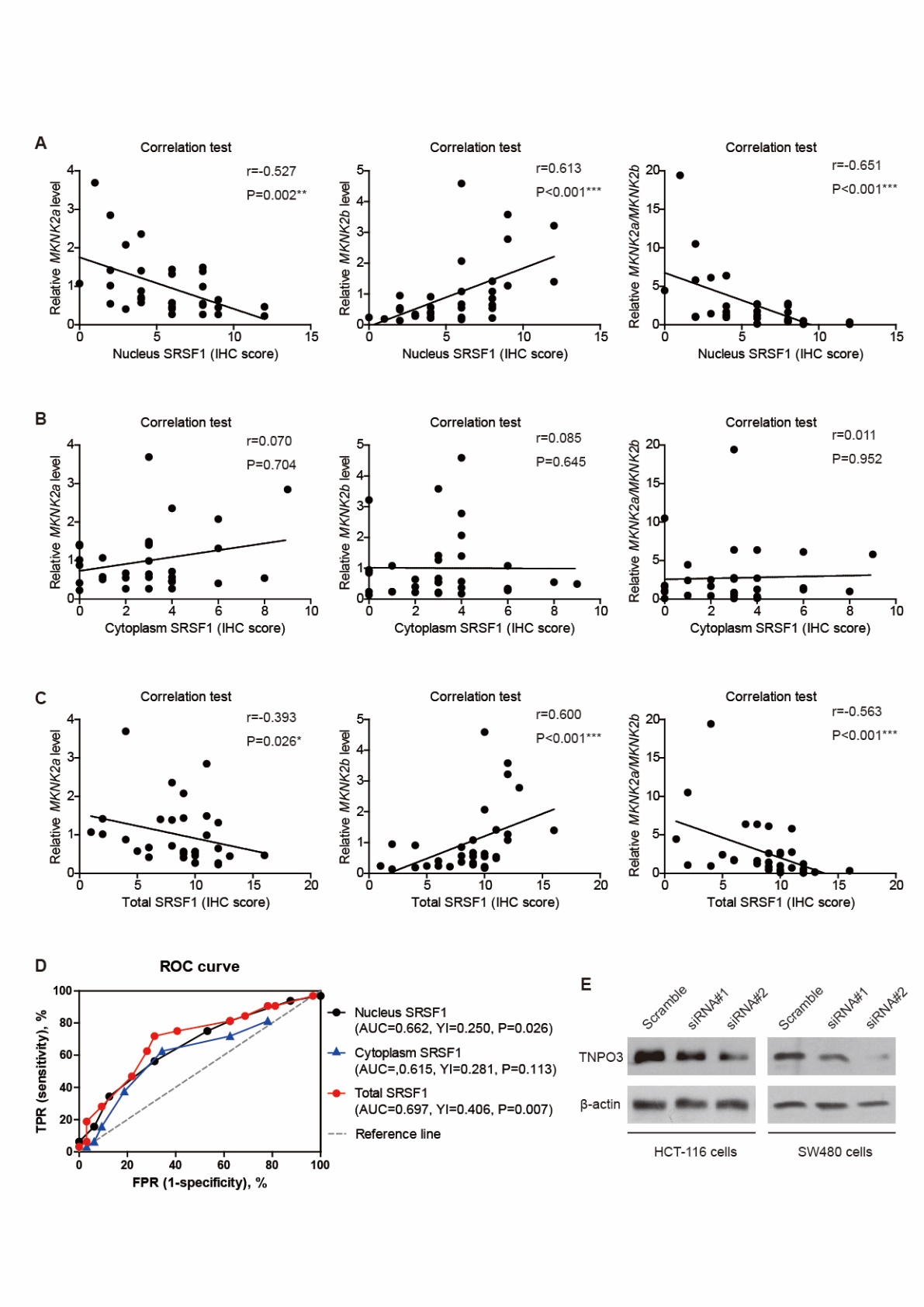


**Figure S3. Correlations between SRSF1 protein expression and *MKNK2* alternative splicing in CAC tissues.**

Correlations between total SRSF1 level (A), nucleus SRSF1 level (B), cytoplasm SRSF1 level (C) with *MKNK2a, MKNK2b*, and *MKNK2a/2b* in clinical specimens (n=32). Data were analyzed by Spearman correlation test. (D) ROC curves of SRSF1 IHC results were plotted. The area under the ROC curves were 0.662, 0.615 and 0.697 for nucleus SRSF1, cytoplasm SRSF1, and total SRSF1, respectively. The P value for the curves were 0.026, 0.113, and 0.007, respectively. According to the ROC curves, patients were divided into high nucleus SRSF1 group with nucleus IHC score >5, low nucleus SRSF1 group with nucleus IHC score ≤5. Similarly, patients were divided into high cytoplasm SRSF1 group with cytoplasm IHC score >3, low cytoplasm SRSF1 group with cytoplasm IHC score ≤3. Patients were additionally divided into high total SRSF1 group with total IHC score >7, low total SRSF1 group with total IHC score ≤7. (E) Silencing efficiency of TNPO3-siRNAs were tested by immunoblotting in HCT-116 and SW480 cells, respectively. We chose siRNA#2 for further experiments due to its better silencing efficiency.


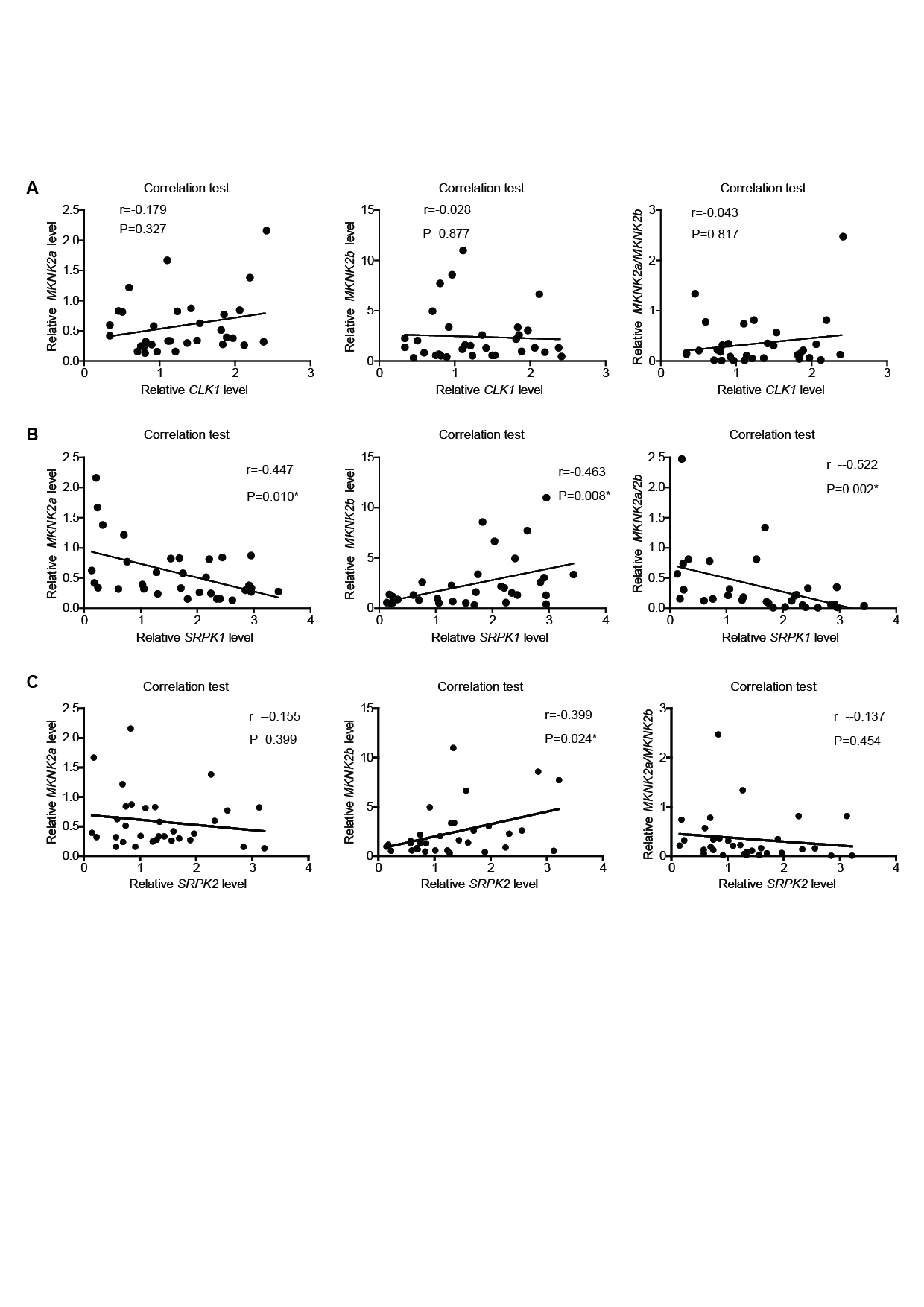


**Figure S4. Correlations between upstream kinases and *MKNK2* alternative splicing in CAC tissues.**

(A) Correlations between *CLK1* level, *SRPK1* level (B), *SRPK2* level (C) with *MKNK2a, MKNK2b*, and *MKNK2a/2b* in clinical specimens (n=32) by RT-qPCR assays. Data were analyzed by Spearman correlation test.


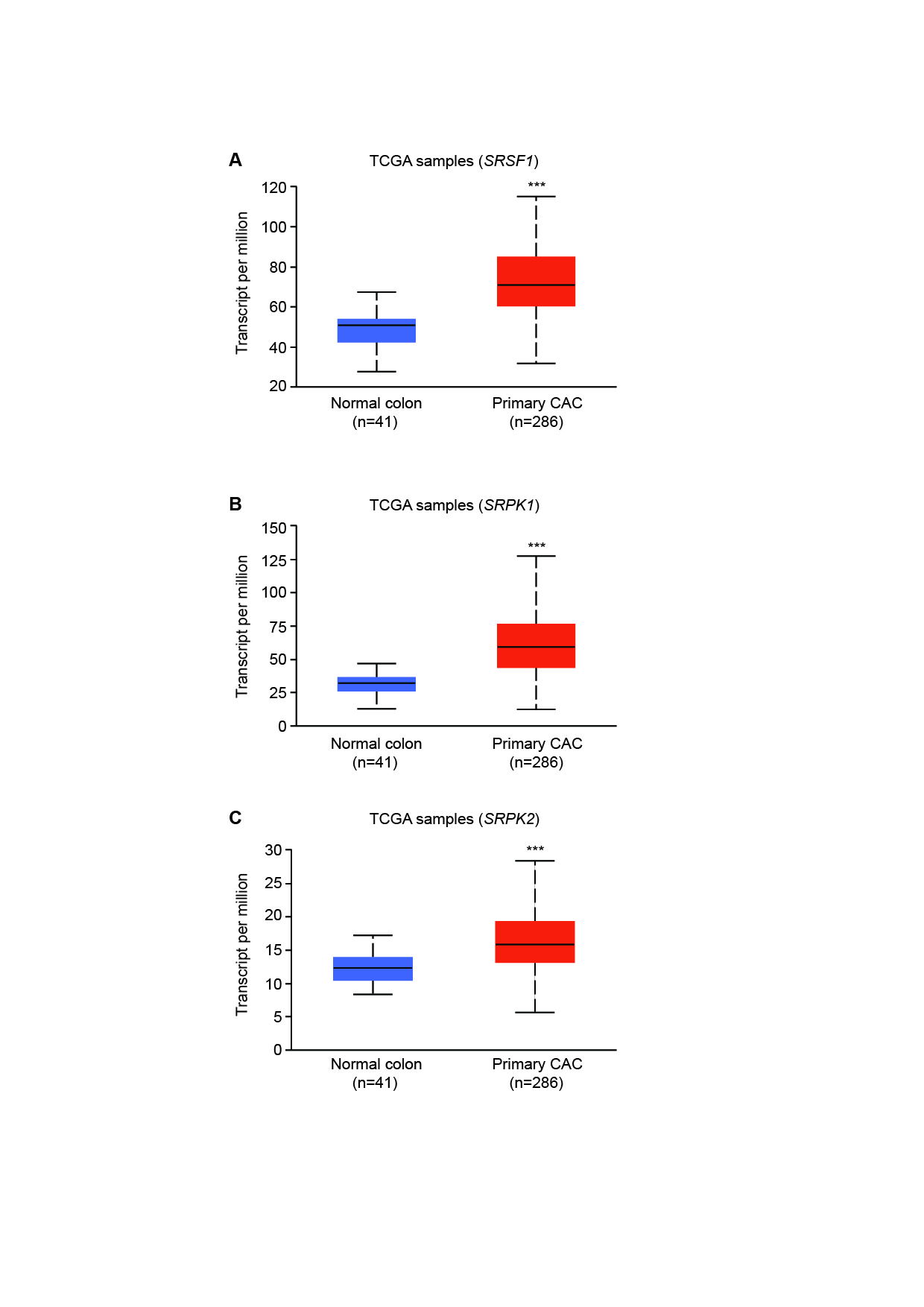


**Figure S5. SRSF1, SRPK1, and SRPK2 are upregulated in CAC tissues.**

Comparisons of *SRSF1* (A), *SRPK1* (B), *SRPK2* (C) between normal colons (n=41) and primary colon adenocarcinomas (n=286) using TCGA RNA-Seq data.


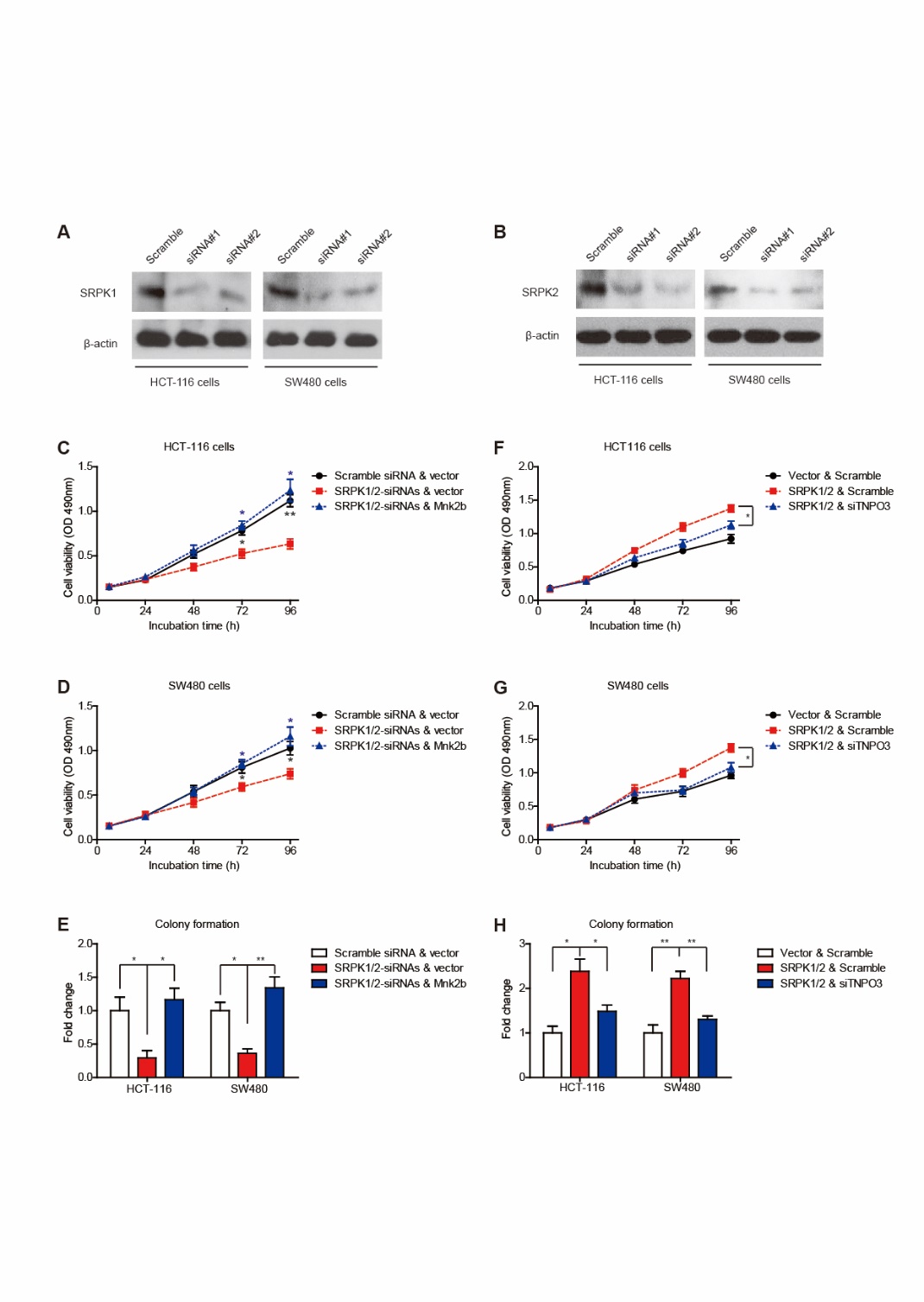


**Figure S6. Effects of SRPKs on cell proliferation can be altered by Mnk2b and TNPO3.**

Silencing efficiencies of different siRNAs targeting SRPK1 (A) and SRPK2 (B) were tested by western blotting, we chose SRPK1-siRNA#1 and SRPK2-siRNA#1 for further experiments due to their better silencing effects.

(C) HCT-116 cells were divided into three groups, i) transfected with scramble siRNA and pCDNA-vector; ii) transfected with SRPK1-siRNA, SRPK2-siRNA, and pCDNA-vector; iii) transfected with SRPK1-siRNA, SRPK2-siRNA, and pCDNA-Mnk2b. The proliferation curves of the three groups were plotted by MTT method, indicating that MNK2b transfection rescued proliferation capacity of the SRPK1/2-knockdown cells. P value was based on unpaired Student’s t-test comparing to cells transfected with SRPK/2-siRNAs & vector.

(D) Similar experiments were conducted on SW480 cells.

(E) Colony formation assay was performed for the three cell groups in both HCT-116 and SW480 cells as described above.

(F) HCT-116 cells were divided into three groups, i) transfected with scramble siRNA and pCDNA-vector; ii) transfected with scramble siRNA, pcDNA-SRPK1, and pcDNA-SRPK2; iii) transfected with TNPO3-siRNA, pcDNA-SRPK1, and pcDNA-SRPK2. The proliferation curves of the three groups were plotted by MTT method, indicating that TNPO3-siRNA transfection attenuated proliferation capacity of the SRPK1/2-overexpressing cells. P value was based on unpaired Student’s t-test.

(G) Similar experiments were conducted on SW480 cells.

(H) Colony formation assay was performed for the three cell groups in both HCT-116 and SW480 cells as described in (F).


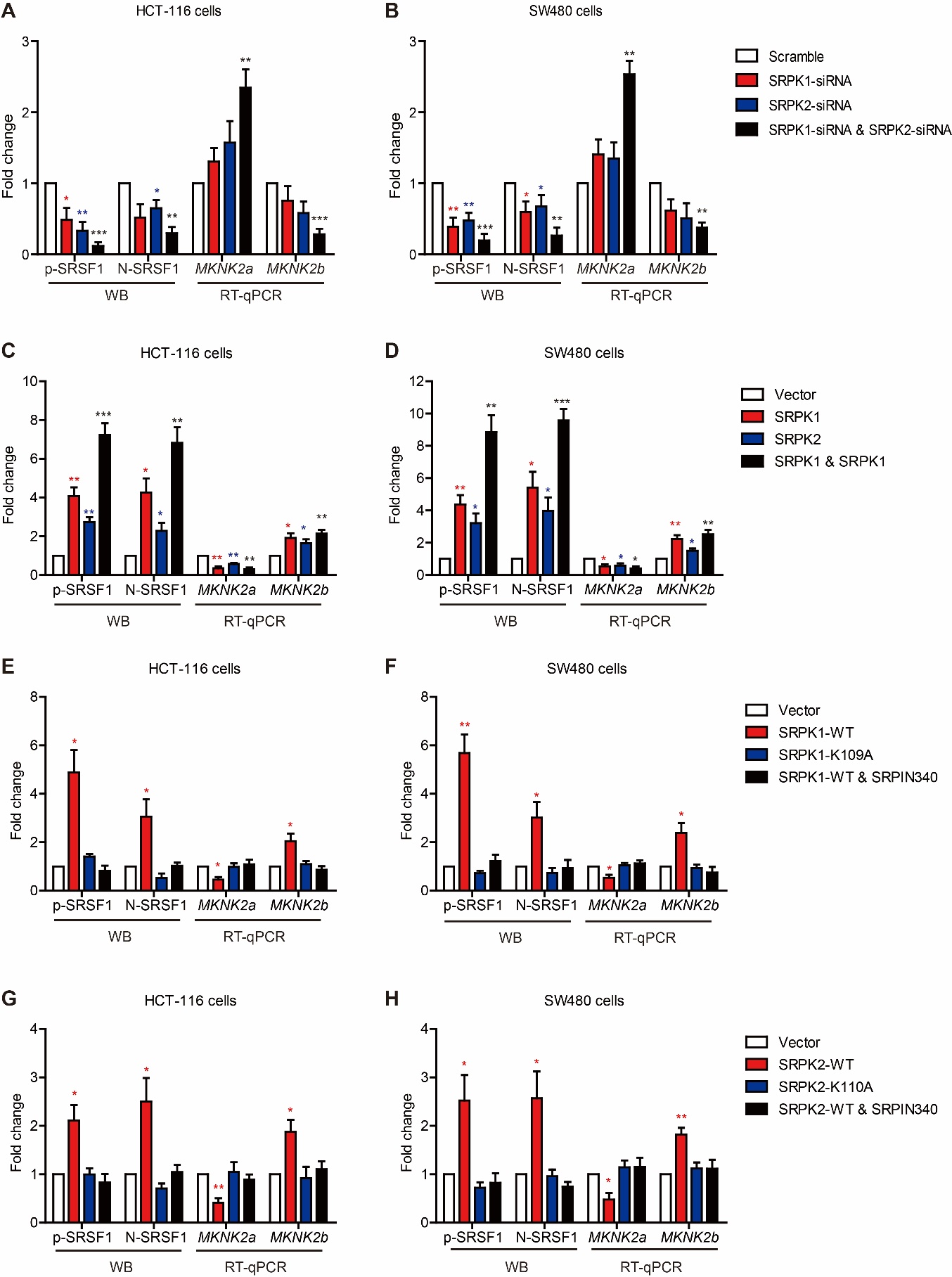


**Figure S7.** **SRPKs modulate SRSF1 phosphorylation, nucleus transportation, and MKNK2 alternative splicing.**

(A, B) Knockdown of either SRPK1 or SRPK2 attenuated phosphorylation of SRSF1 (p-SRSF1) and reduced nucleus SRSF1 (N-SRSF1) level. RT-qPCR results showed a positive correlation between *MKNK2b* and SRPKs. Furthermore, simultaneously knockdown of SRPK1 and SRPK2 exhibited the most significant effects, indicating a synergic function of SRPK1 and SRPK2 on modulating SRSF1-dependent *MKNK2* splicing. P value was based on unpaired Student’s t-test comparing with scramble group.

(C, D) Overexpressing SRPKs increased phosphorylation of SRSF1 and promoted its nucleus translocation according to immunoblotting results. RT-qPCR experiments showed that SRPKs was negatively correlated with *MKNK2a* level while positively correlated with *MKNK2b* level. RT-qPCR were also conducted to quantitatively analyze changes of *MKNK2a* and *MKNK2b*, which showed consistent trends with WB data. P value was based on unpaired Student’s t-test comparing with vector group.

(E, F) SRPIN340 treatment towards SRPK1-overexpressing cells abolished the effects above. Consistently, the kinase dead (KD) mutant SRPK1-K109A, lost effects on modulating SRSF1 phosphorylation, subcellular location, and *MKNK2a-MKNK2b* switch. The data were obtained by western blotting and RT-qPCR. P value was based on unpaired Student’s t-test comparing with vector group.

(G, H) SRPIN340 treatment towards SRPK2-overexpressing cells also abolished its effects on modulating SRSF1 phosphorylation, subcellular location, and *MKNK2a-MKNK2b* switch. The KD mutant of SRPK2, namely SRPK2-K110A, showed similar effects with SRPIN340. P value was based on unpaired Student’s t-test comparing with vector group.


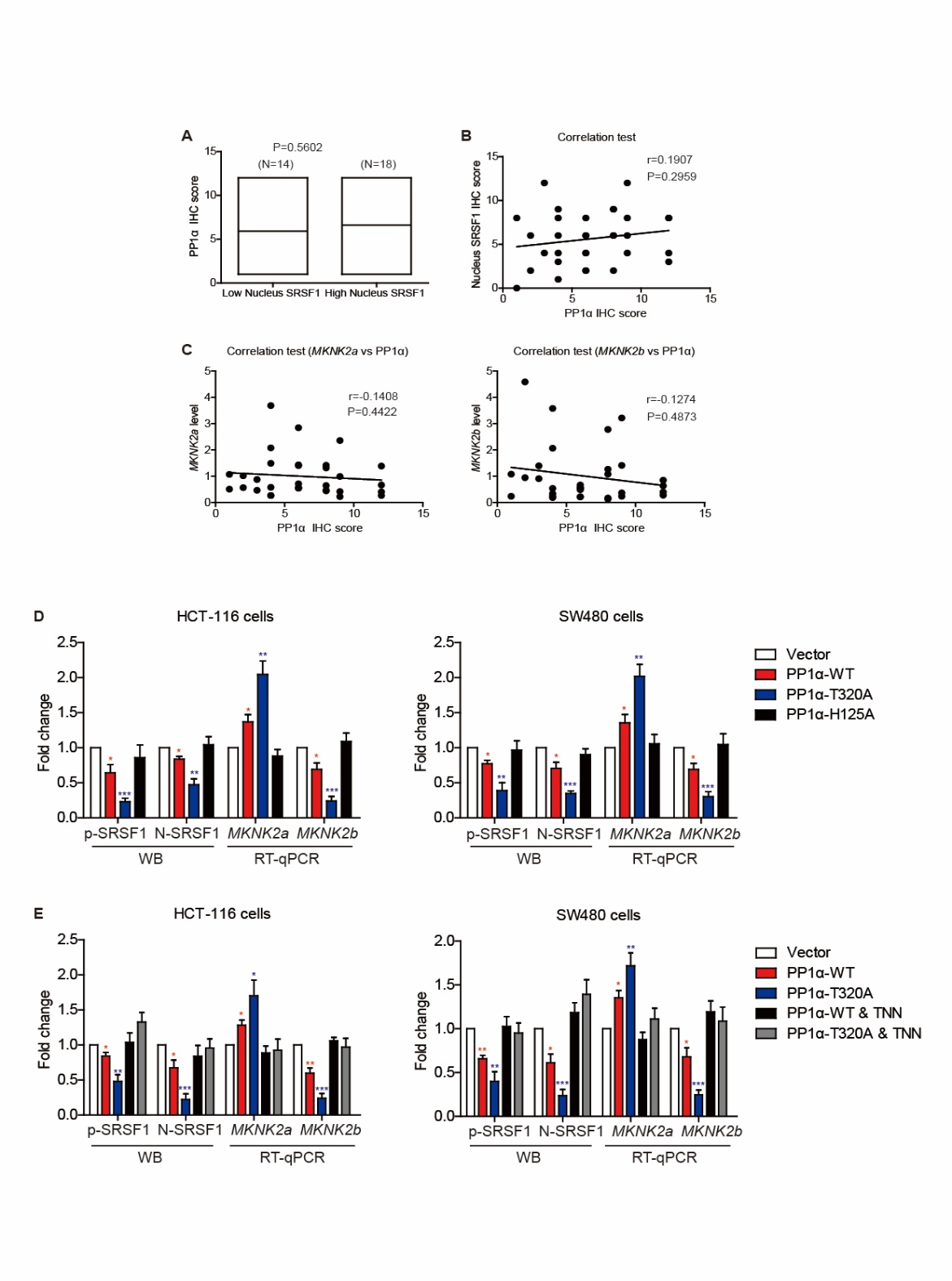


**Figure S8. Functions of PP1α on modulating SRSF1-dependent *MKNK2* splicing.**

(A) Patients were divided into two subgroups depending on the nucleus level of SRSF1, namely low nucleus SRSF1 group and high nucleus SRSF1 group. There was no statistically significant difference of PP1α IHC results between the two groups. P value was based on unpaired Student’s t-test.

(B) The correlation between PP1α and nucleus SRSF1 level was evaluated based on their IHC score using Spearman correlation test, which showed no significant correlation (n=32).

(C) Protein level of PP1α has no correlation with MKNK2 variants in clinical specimens (n=32).

(D) The phosphor-SRSF1 (p-SRSF1) and nucleus SRSF1 (N-SRSF1) levels were semi-quantified corresponding to Fig. 6J. RT-qPCR were also conducted to quantitatively analyze changes of MKNK2a and MKNK2b, which showed consistent tendency with RT-PCR data in Fig. 6J. P value was based on unpaired Student’s t-test comparing with vector group.

(E) The p-SRSF1 and nucleus SRSF1 levels were semi-quantified corresponding to Fig. 6K. RT-qPCR were also conducted to quantitatively analyze changes of MKNK2a and MKNK2b, which showed consistent tendency with RT-PCR data in Fig. 6K. P value was based on unpaired Student’s t-test comparing with vector group.


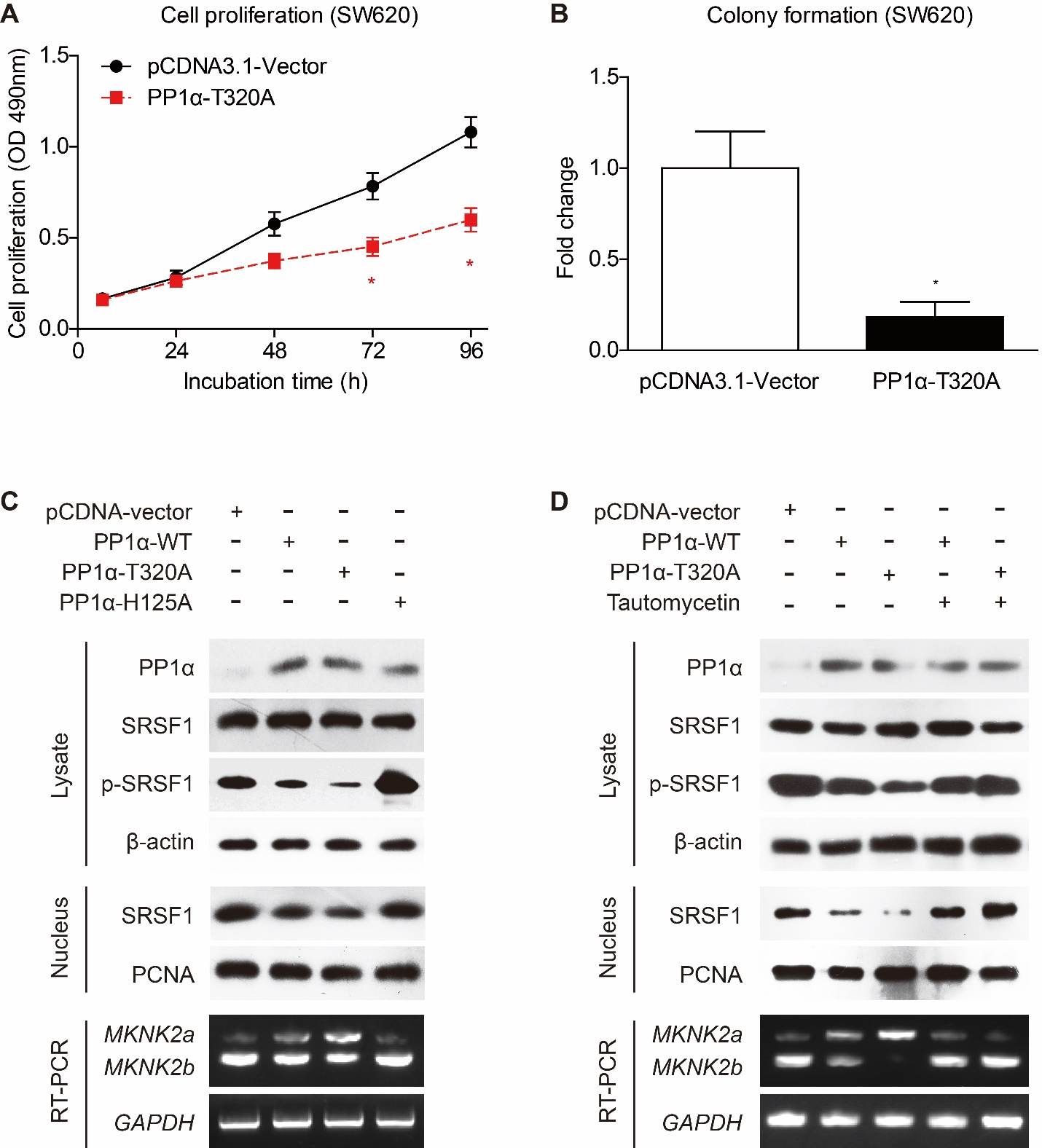


**Figure S9. Functions of PP1α on modulating proliferation and SRSF1-dependent *MKNK2* splicing in metastatic SW620 cells.**

(A) Proliferation capacity of SW620 cells was impaired by overexpressing pcDNA-PP1α-T320A, a constitutively active PP1α construct, as revealed by MTT assays. P value was based on unpaired Student’s t-test.

(B) Colony formation assay was conducted for the cells described above, further indicating that PP1α-T320A may attenuate SW620 proliferation. P value was based on unpaired Student’s t-test.

(C) SW620 cells were transfected with three different PP1α constructs, including PP1α-WT, PP1α-T320A (constitutively active mutant), and PP1α-H125A (kinase-dead mutant) using pcDNA-vector as control. The levels of phosphorylated SRSF1, nucleus SRSF1 were measured by western blotting, and the *MKNK2* splicing were evaluated by RT-PCR (lower panel).

(D) SW620 cells were transfected with PP1α-WT or PP1α-T320A with/without tautomycetin treatment. The levels of phosphorylated SRSF1, nucleus SRSF1 were measured by western blotting, and *MKNK2* splicing were evaluated by RT-PCR (lower panel).
